# The balance between R-loops and RNAPII occupancy determines the outcome of transcription–replication conflicts

**DOI:** 10.1093/nar/gkag1139

**Published:** 2026-11-11

**Authors:** Ioannis Tsirkas, Clare S K Lee, Daniel Dovrat, Neha Singh, Zohar Paleiov, Maxime Lalonde, Atiqa Sajid, Tobias Straub, Sophia G Sergi, Fabio Bento, Brian Luke, Amir Aharoni, Stephan Hamperl

**Affiliations:** Chromosome Dynamics and Genome Stability, Institute of Epigenetics and Stem Cells, Helmholtz Munich, Munich 81377, Germany; Chromosome Dynamics and Genome Stability, Institute of Epigenetics and Stem Cells, Helmholtz Munich, Munich 81377, Germany; Department of Life Sciences, Ben-Gurion University of the Negev, Be’er Sheva 84105, Israel; Department of Life Sciences, Ben-Gurion University of the Negev, Be’er Sheva 84105, Israel; Department of Life Sciences, Ben-Gurion University of the Negev, Be’er Sheva 84105, Israel; Chromosome Dynamics and Genome Stability, Institute of Epigenetics and Stem Cells, Helmholtz Munich, Munich 81377, Germany; Chromosome Dynamics and Genome Stability, Institute of Epigenetics and Stem Cells, Helmholtz Munich, Munich 81377, Germany; Core Facility Bioinformatics, Biomedical Center (BMC), LMU Munich, Munich 80539, Germany; Institute of Developmental Biology and Neurobiology (IDN), Johannes Gutenberg Universität, Mainz 55128, Germany; Institute of Molecular Biology (IMB), Mainz 55128, Germany; Institute of Developmental Biology and Neurobiology (IDN), Johannes Gutenberg Universität, Mainz 55128, Germany; Institute of Molecular Biology (IMB), Mainz 55128, Germany; Institute of Developmental Biology and Neurobiology (IDN), Johannes Gutenberg Universität, Mainz 55128, Germany; Institute of Molecular Biology (IMB), Mainz 55128, Germany; Department of Life Sciences, Ben-Gurion University of the Negev, Be’er Sheva 84105, Israel; Chromosome Dynamics and Genome Stability, Institute of Epigenetics and Stem Cells, Helmholtz Munich, Munich 81377, Germany

## Abstract

Maintaining genome integrity requires the precise coordination of replication and transcription. While R-loops are known to cause transcription–replication conflicts (TRCs), the precise nature of the barrier, whether it is the RNA:DNA hybrid itself, the stalled RNA Polymerase II (RNAPII), or a combination of both, remains elusive. Using live-cell imaging in yeast to monitor a single R-loop prone locus in real time, we demonstrate that wild-type replisomes traverse R-loop enriched transcription units with high efficiency. We find that while R-loops initially repress transcription, replisome passage prompts a robust recovery of transcription activity. Indeed, we discover that R-loops serve a critical regulatory function by suppressing excessive RNAPII occupancy. In an RNAPII mutant with high DNA affinity, the enzymatic removal of R-loops leads to a pile-up of RNAPII molecules, creating a barrier to replication, more severe than the R-loops themselves. Thus, our findings reveal a dual nature of R-loops: while they can be obstacles and repress transcription, they can also serve as a physiological buffer that prevents the formation of high-density RNAPII ‘trains’. These findings establish that the precise regulation of both R-loop levels and RNAPII density is critical for preventing harmful TRCs and maintaining genome stability.

## Graphical Abstract

**Graphical Abstract:**
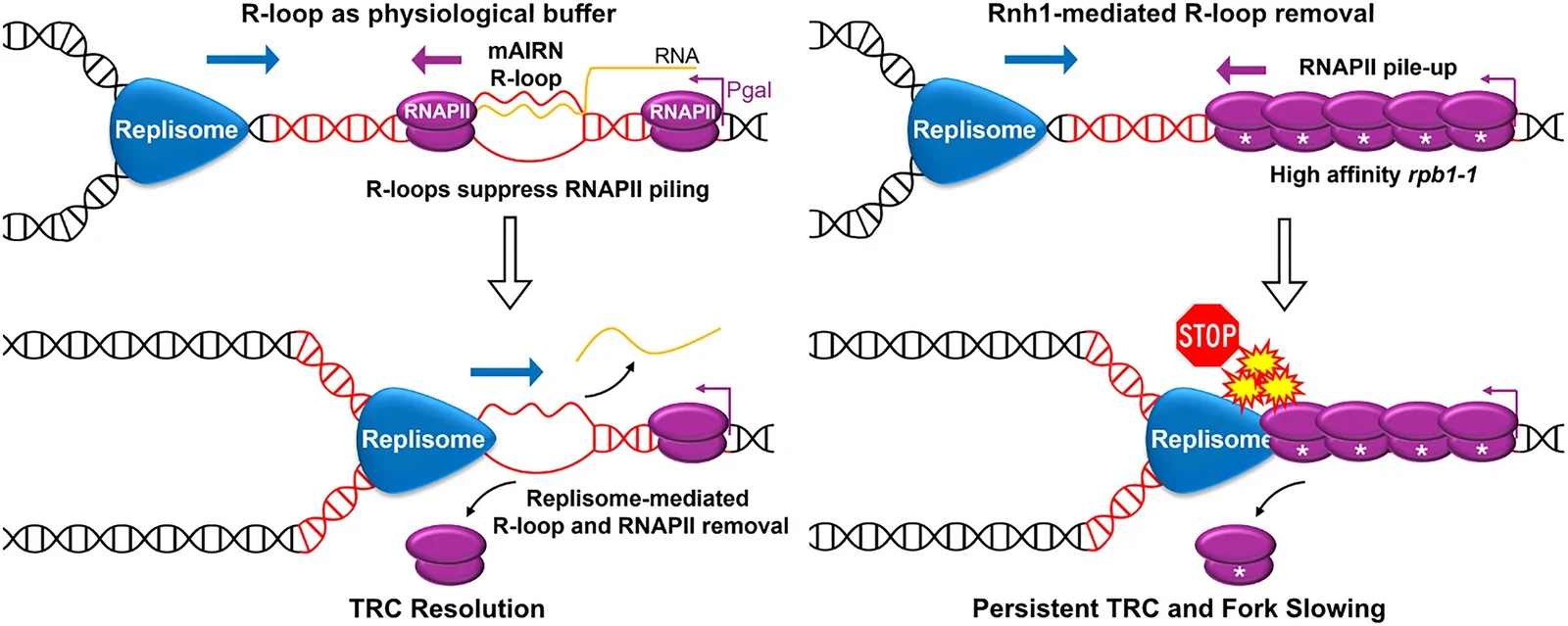

## Introduction

R-loops are non-B DNA structures formed co-transcriptionally when the newly-synthesized RNA strand anneals with its complementary DNA strand, displacing the single-stranded coding DNA [1]. While they facilitate physiological processes like transcription termination and immunoglobulin class switching, their persistence causes DNA damage hotspots, contributing to genomic instability and mutagenesis [2–4]. Recent discoveries have connected R-loop persistence with several human diseases such as oncogenesis and neurological disorders, establishing their clinical significance as diagnostic tool and potential biomarkers [5, 6]. R-loop mapping studies have revealed hundreds to thousands of R-loop prone genomic sequences [7–9]. Given the abundance of R-loops and the inherent risk they pose to genomic stability, how cells precisely orchestrate their formation with the advancing transcription and replication machineries remains a fundamental, yet unresolved, question.

DNA replication and transcription machineries ensure faithful duplication of the genome and proper gene expression, respectively. However, both machineries share the same DNA template. During S-phase, a replisome may confront multiple RNA polymerases (RNAP) occupying highly transcribed genes, leading to transcription–replication conflicts (TRCs) [10–12]. Although the two processes are generally segregated both spatially and temporally [13], it has been shown that TRCs can occur frequently in eukaryotic cells [14, 15]. If not resolved, they can result in replication fork stalling or collapse, causing genomic instability and increased frequency of DNA double-strand breaks [16–18], especially in the event where the transcribing RNAP is facing the oncoming replisome head-on (HO) [19, 20]. Co-directional (CoD) conflicts are considered less detrimental, but can still interfere with replication fork progression, particularly when replication forks are challenged with more persistent transcriptional blocks such as R-loops [20–22]. Thus, understanding how cells avoid and overcome such hazardous R-loop dependent TRCs is of paramount importance for deciphering the pathways how they contribute to genome instability.

Apart from their impact on replisome progression, R-loops were shown, both *in vitro* and *in vivo*, to impede transcription by interfering with RNAPII progression, resulting in early transcription termination [23–25]. Arrest of RNAPII’s progression by R-loops can eventually lead to their eviction and sequential degradation [26, 27]. Furthermore, thousands of genes have been identified with skewed GC content at their 3′ ends [28], consistent with a role of R-loops in transcription termination. Thus, R-loops can have both a negative impact on genomic stability and, on the other hand, regulatory roles during genome duplication and transcription.

Considering the disruptive potential of R-loops, cells have evolved an extensive plethora of protein factors to counteract RNA:DNA hybrid accumulation. This includes RNA packaging and processing machineries that prevent the initial formation of RNA–DNA hybrids [29, 30], proteins that enzymatically degrade and remove RNA:DNA hybrids from the genome, such as the family of Ribonuclease H (RNH) enzymes [31] or RNA:DNA helicases designated to unwind R-loops and maintain unhindered replisome progression and gene transcription [32–34]. This redundancy suggests that R-loop levels need to be tightly regulated to preserve proper gene expression, replisome progression, and cellular function [35]. One main limitation in studying the interplay between R-loops, transcription, and replication is the requirement of tools for the simultaneous measurement of fork progression and transcription dynamics at an inducible R-loop gene in a single cell cycle in live cells.

Here, we took advantage of a previously established system for the real-time monitoring of transcription and replication at an individual locus in single live cells [36]. By inserting the inducible, highly R-loop prone mouse antisense of IGF2R non-protein coding RNA (mAIRN) sequence in the path of a single replisome, we found a surprising reciprocal relationship: while R-loop formation initially strongly represses mAIRN transcription, the passage of the replisome triggers a robust recovery of transcriptional activity. This observation suggests a replisome-dependent regulation of transcriptional activity at R-loop forming genes. Moreover, we provide evidence that R-loops have a beneficial role to limit RNAPII density and prevent the formation of dense transcriptional clusters that would otherwise cause replication fork stalling. Overall, our results reveal that R-loop levels and RNAPII density are interconnected parameters and cells utilize this balance to regulate transcription dynamics and ensure the suppression of harmful TRCs.

## Materials and methods

### Reagents and biological materials

A detailed list of yeast and bacterial strains (Supplementary Table S1), plasmids (Supplementary Table S2), oligonucleotides (Supplementary Table S3), reagents, commercial kits, and assays (Supplementary Table S4), as well as software and algorithms (Supplementary Table S5) used in this study can be found in the Supplementary data.

### *Saccharomyces cerevisiae* strains generation

All yeast strains used in this study were generated on the background of W1588, which is identical to W303 but with a wild-type copy of Rad5, MATa *S. cerevisiae* strain, integrated with *lacO*x256 and *tetO*x224 arrays at *chrIV*:332960 and *chrIV*:352560, respectively, adjacent to *ARS413*. LacI-HaloTag and tetR-tdTomato fusion proteins are expressed in the nucleus to visualize the arrays as distinct fluorescent foci [36]. The mAIRN sequence (1318 bp) was expressed from the unidirectional GAL10 promoter (Pgal, 404 bp) and labelled with 14xPP7 (857 bp). Then, this mAIRN reporter cassette was flanked by CUT60 and ADH1 terminator sequences and the whole cassette was amplified and inserted between the two arrays at *chrIV*:336186 in either CoD or HO orientation relative to replisome emerging from *ARS413*. The mAIRN reporter cassette integration was performed using a marker-free CRISPR–Cas9 approach, targeting *natMX* antibiotic cassettes with specific guide RNAs (gRNAs) [37].

For Rnh1 overexpression (OE), RNH1orf was expressed by the Pgal (454 bp) and three copies of HAtag were fused to its C-terminus, followed by CYC1 terminator, in a URA3-integrative plasmid. The generation of *rnh1rnh201*ΔΔ and *mph1*Δ yeast strains was performed by replacing the respective genes with *natMX, hphMX*, or *kanMX* antibiotic cassettes. The *sen1-3* mutant (W773A, E774A, W777A) strains [38] were generated with CRISPR–Cas9, using the gRNA: TAAAATTCTGGGAATCATGT targeting the SEN1 gene in the mutated region, using a DNA donor containing the mutation with 80 bp homology to the genome on each side of the targeted region. The *rpb1-1* mutant (G4622A) strains [39] were generated with CRISPR–Cas9, as described before [36]. MNase fusion of RPB2 was performed by amplification of the MNase-3xHA-*kanMX* cassette from a plasmid and integration in the C-terminus of RPB2 gene, excluding the native termination codon. All integrations, replacements, and mutations were validated by polymerase chain reaction (PCR) and Sanger sequencing.

### Microscopy

Yeast cells were grown overnight in synthetic complete (SC) medium containing 2% raffinose (RAF) at 30°C. Yeast cultures were diluted at OD_600_ = 0.2 and SiR-HALO dye was added to a final concentration of 800 nM. One hour following SiR-HALO dye addition, 10 μg/ml α-factor was added to arrest the cells in G1 phase, and the cultures were incubated for two additional hours. Cells were then immobilized on microscopy slide chambers (Ibidi) coated with 2 mg/ml concanavalin A for 10–15 min and washed thoroughly from α-factor and SiR-HALO dye with warm SC medium containing 2% RAF prior to microscopy experiments. For the induction of mAIRN transcription, 2% galactose (GAL) was added to the culture media 2 h before and during the microscope experiment. Live-cell imaging of the cells was performed on an Axio Observer inverted wide-field microscope (Zeiss) with a Colibri 7 LED light source, at 1-min intervals for 4 h at 30°C, using a 63× oil objective (NA = 1.4) in 3D (8 z-sections, 0.8 μm apart). LacI-Halo-SiR and tetR-tdTomato were excited with 650 and 561 nm illumination, respectively. PCP-Envy was excited with 488 nm illumination.

### Live-cell imaging quantification

Time-lapse measurements were collected with ZEN3.0 and analysed using a custom-made computational pipeline developed specifically for the analysis of replication rates and transcription dynamics during replisome progression by the Aharoni group (Transcriptomatic5). The MATLAB-based pipeline identifies, tracks, and quantifies the LacI-Halo-SiR, tetR-tdTomato, and PCP-Envy dots in each cell. Proper identification of the PCP-Envy dot that corresponds to the transcription site is validated by ensuring colocalization with the lacI-Halo-SiR and tetR-tdTomato dots, and the PCP-Envy background signal is subtracted to quantify transcription site intensity. Time axes per cell are normalized so that the timespan 0 to 1 corresponds to the replication time of the specific cell. Quantification results for multiple cells in each strain are averaged, while normalizing the time axis for each individual cell according to its replication rate, such that the average transcriptional intensity is calculated relative to the location of the replisome along the chromosome, finally representing the median replication time per yeast strain under induced conditions (+GAL 2%). Swarm plots and violin plots were plotted in Python.

### Western blot analysis 

For the detection of Rnh1-3xHA or Rpb2-MNase-3xHA expression by western blot, 5 ml of yeast cells were grown until OD_600_ ∼0.5 in 2% RAF. For induction of PP7-mAIRN and Rnh1 OE, 2% GAL was added in the media and cells were grown for an additional 2 h (OD_600_ ∼1). Two to three millilitres of cell suspensions were transferred into separate 1.5-ml tubes and centrifuged at >20 000 × *g* for 1 min. The supernatants were discarded and cell pellets were snap-frozen in liquid N_2_ and stored in −80°C overnight.

The next day, the pellets were resuspended in 500 μl ice-cold ddH_2_O and 75 μl NaOH/β-mercaptoethanol (β-ME) solution [1.85 M NaOH and 7.5% (v/v) β-ME]. The cell solutions were vortexed for 4 s and incubated on ice for 15 min. Then, 75 μl of 55% trichloroacetic acid was added, the tubes were vortexed for 4 s, and incubated on ice for 10 min. Lysed cells were centrifuged for 10 min at >20 000 × g and at 4°C and the supernatant was discarded. Finally, tubes were centrifuged for another 30 s for 10 min at >20 000 × *g* at 4°C to collect and discard completely the residual liquid. Pellets were resuspended with 30–40 μl of high urea protein loading buffer [8 M urea, 5% sodium dodecyl sulphate (SDS), 200 mM Tris–HCl, pH 6.8, 1 mM ethylenediaminetetraacetic acid (EDTA), 0.1% (w/v) bromophenol blue]. If the suspensions turned green or yellow colour, 1 μl of 1 M Tris–HCl, pH 8 was added. Proteins were denatured at 65°C for 10 min with mild shaking.

Four to ten microlitres of protein lysates were loaded and run on a 10% sodium dodecyl sulphate–polyacrylamide gel electrophoresis (SDS–PAGE) gel for 1.5–2 h at 120 V. Separated protein bands were transferred to a PVDF membrane followed by blocking with PBST + 3% bovine serum albumin (BSA) + 5% skim milk overnight. The membrane was washed with PBST once for 5 min, and was incubated with a rat anti-HA antibody conjugated with HRP enzyme (1:2000) (Cat# 12013819001) in PBST + 3% BSA for 1 h. The membrane was washed three times with PBST and HA-labelled Rnh1 or Rpb2 were detected with the EZ-ECL Chemiluminescence detection kit (Thermo Fisher Scientific) according to the manufacturer’s protocol. Detection of RNAPII bands was performed with a primary rabbit anti-RNAPII antibody (Cat# ab193468) (1:2000) and a secondary goat-anti-rabbit antibody conjugated with HRP (Cat # G21234) (1:10 000).

### DRIP-qPCR

DRIP in yeast strains was performed as previously described [40] with slight modifications. Yeast cells were grown overnight in 100 ml yeast peptone with 2% RAF (YPR) until OD_600_ ∼0.5. When specified, 2% GAL was added in the media and the cultures were grown for another 2 h, until OD_600_ ∼1. Cell cultures were harvested in 2 × 50 ml falcon tubes by centrifugation at 4000 × *g* at 4°C for 5 min and were washed twice with 25 ml cold ddH_2_O. Then, yeast pellets were resuspended in 2.4 ml freshly prepared Spheroplasting buffer [1 M sorbitol, 2 mM Tris–HCl, pH 8.0, 100 mM EDTA, pH 8.0, 0.1% (v/v) β-ME] supplemented with 40U Zymolyase 20T (Cat# 9329.1) per sample (40 μl of 50 mg/ml, 2000 U/mg) in 2 × 2 ml tubes and spheroplasted for 30–40 min at 30°C at 500 rpm. Spheroplasts were centrifuged at 4500 × *g* at 4°C for 5 min, supernatants were carefully removed, and spheroplast pellets were washed on top with 500 μl cold ddH_2_O without resuspending. Tubes were briefly spin down to remove the ddH_2_O, spheroplasts were resuspended with 565 μl Solution 1 [0.8 mM GuHCl, 30 mM Tris–HCl, pH 8.0, 30 mM EDTA pH 8.0, 5% (v/v) Tween 20, 0.5% (v/v) Triton X-100] and split samples were combined in 1.5-ml tubes and were treated with 20 μl RNase A (10 mg/ml) and 150 μl Proteinase K (10 mg/ml) for 30 min at 37°C and 1 h at 50°C, respectively, at 500 rpm. Samples were centrifuged at 4500 × *g* at 4°C for 5 min and supernatants were carefully transferred in fresh 1.5-ml tubes with 1 ml cut tips. Eight-hundred microlitres cold phenol–chloroform–isoamylalcohol were added, and samples were vortexed for at least 20–30 s and centrifuged at full speed at 4°C. Supernatants were carefully transferred into fresh 2 ml tubes with 1 ml cut tips and genomic DNA was precipitated with 800 μl Isopropanol by gently inverting several times. Precipitated DNA samples were transferred with 1 ml cut tip in a fresh 1.5-ml DNA-low-binding tube, washed twice with 800 μl of 70% ethanol, and allowed to air-dry for 15 min before resuspending with 150 μl TE buffer (10 mM Tris–HCl, pH 8.0, 1 mM EDTA) by shaking at 500 rpm at 25°C. Genomic DNA containing RNA:DNA hybrids was digested overnight at 37°C with a restriction enzyme cocktail (2 μl BsrGI, 2 μl EcoRI, 2 μl HindIII, 2 μl PmlI, 2 μl SacI, and 2 μl XbaI with NEB rCutsmart Buffer) at final volume of 250 μl.

Next day, extra 1 μl of each restriction enzyme were added, samples were incubated for extra 2–3 h at 37°C, and DNA was isolated with another round of phenol–chloroform–isoamylalcohol extraction and resuspended in 50 μl TE buffer. DNA was quantified using a NanoDrop and 1–4 μg were further diluted in a total of 500 μl TE buffer used for DRIP. For *in vitro* Rnase H treatment, 3–4 μg of restriction enzyme digested DRIP DNA samples were incubated overnight at 37°C with 1 μl RNase H (NEB Cat# M0523) in 1× RNase H reaction buffer at final volume of 100 μl, followed by phenol–chloroform–isoamylalcohol extraction and ethanol precipitation as outlined above. DNA was resuspended in 50 μl TE buffer and further diluted in a total of 500 μl TE buffer as the regular DRIP samples. Fifty microlitres of this solution (10%) were transferred in a separate tube and were used as Input (IN), while the remaining 450 μl were mixed with 50 μl of 10× Binding Buffer [100 mM NaPO_4_, pH 7.0, 1.4 M NaCl, 0.5% (v/v) Triton X-100] and 4–7 μl anti-RNA:DNA hybrid antibody (Ab), and were incubated overnight at 4°C on a rotator mixer.

Next day, 50 μl of Pierce™ Protein A/G agarose beads were washed thrice with 1× Binding Buffer diluted in TE buffer, resuspended with 50 μl TE buffer, mixed with the immunoprecipitated DNA samples, and incubated at 4°C on a rotator mixer for 2 h. Then, samples were centrifuged at 400 × *g* at 4°C for 2 min, supernatants were discarded, and the beads were washed three times with 1× Binding Buffer. After the last wash, agarose beads were resuspended with 100 μl TE buffer and were incubated with 7 μl Proteinase K (10 mg/ml) at 55°C at 800 rpm for 45 min. One-hundred microlitres IRN buffer (50 mM Tris–HCl, pH 8.0, 20 mM EDTA, 500 mM NaCl) and 200 μl phenol–chloroform–isoamylalcohol were added in IP and IN samples, and the tubes were vortexed vigorously. The aqueous phase was transferred in a fresh 1.5-ml DNA-low-binding tube and DNA was precipitated with 600 μl of 100% EtOH and 2 μl glycogen (5 mg/ml) for 1 h at −20°C. After centrifugation at full speed at 4°C for 30 min, supernatants were discarded, DNA pellets were washed twice with 70% EtOH, and finally resuspended in 50 μl TE buffer. Enrichment over IN of the desired sequences was estimated with qPCR, using the iTaq Universal SYBR Green Supermix (Bio-Rad) with the indicated primers (Supplementary Table S3). Data were plotted with GraphPad Prism 9.

### Yeast cell growth for chromatin immunoprecipitation and chromatin endogenous cleavage assay

Yeast cells were grown overnight in 50 ml YPR until OD_600_ ∼0.5, then 2% GAL was added in the media and the cultures were grown for another 2 h, until OD_600_ ∼1. For experiments with cell cycle synchronization, overnight cell cultures were diluted at 50 ml and OD_600_ ∼0.1–0.2 in 250 ml flasks and they were grown until OD_600_ ∼0.5 or 0.6 (∼3 h). Then, 10 μg/ml α-factor was added together with 2% GAL and cells were incubated for another 2 h, until OD_600_ ∼1. Then, cells were split into 50 ml tubes and were centrifuged in a 30°C prewarmed centrifuge for 4 min at 1000 × *g*. The supernatants were discarded and cells of the same strain were collected in a single 50 ml tube. Cells were washed from α-factor twice with 45 ml prewarmed YPR + 2% GAL, and they were resuspended in 100 ml warm YPR + 2% GAL. Forty-five millilitres of yeast cultures were sampled at desired timepoints (10 and 50 min post release into S phase).

### Protein crosslinking for ChIP and ChEC

For crosslinking, 3 ml of 16% formaldehyde (FA) was added dropwise in 45 ml of yeast cultures (final concentration 1%) and cells were fixed in 100-ml flasks for 15 min at 30°C with shaking. FA was quenched with 2.6 ml of 2.5 M glycine (final concentration 128 mM) for 5 min at 30°C. Cells were transferred and harvested in 50 ml tubes (1000 × *g*, 2 min at 4°C), and washed once with ice-cold phosphate buffered saline (PBS). After this step, fixed cells were kept on ice. Washed cells were centrifuged (1000 × *g*, 2 min at 4°C) and cell pellets were resuspended in 1 ml of ddH_2_O and transferred into 1.5-ml tubes. Yeast cells were collected by centrifugation at 20 000 × *g* for 1.5 min at 4°C in a microcentrifuge, the supernatants were discarded, and the pellets were frozen in liquid N_2_ and stored at −80°C for at least overnight.

### RNAPII ChIP-qPCR

ChIP-qPCR experiments were performed as described before [41]. Samples and buffers were kept on ice at all times. Briefly, frozen fixed cell pellets were washed with 500 μl lysis buffer [50 mM HEPES, pH 7.5, 140 mM NaCl, 5 mM EDTA, pH 8, 5 mM EGTA, pH 8, 1% Triton X-100 (w/v), 0.1% DOC (w/v), 1× protease and phosphatase inhibitors] and then they were centrifuged at 20 000 × *g* for 1.5 min at 4°C. The supernatants were discarded and cell pellets were resuspended with 500 μl lysis buffer. Approximately 750 μl precooled glass beads (1 mm) were added to cover the whole suspension and the tubes were filled with lysis buffer. Cells were disrupted on a VXR basic IKA Vibrax orbital shaker at 2200 rpm or an eppendorf ThermoMixerC at 2000 rpm at 4°C for 15 min, three times, with 10 min breaks on ice in between. The bottom and top of the 1.5-ml tube were pierced using a hot needle and placed into a 15-ml tube. After centrifugation (130 × *g*, 2 min at 4°C), the lysates were collected in the 15-ml tubes while the glass beads stayed in the tube. The volume of the suspension was increased to 1 ml with lysis buffer and transferred to a 1-ml Covaris sonication glass tube. Sonication was performed on a Covaris instrument (25 min, peak incident power: 140 W, duty factor: 5%, cycles/burst: 200). Afterwards, the sheared chromatin was cleared by centrifugation (20 min, 20 000 × *g* at 4°C) and the supernatant was transferred to an 1.5-ml DNA-low-binding tube. The solution was split into two aliquots: a total of 90 μl served as IN control, and 810 μl was used for IP.

Forty microlitres protein A magnetic beads were washed thrice with lysis buffer at 4°C and were resuspended with the 810 μl of sheared chromatin. The solution was filled up to 990 μl with lysis buffer, supplemented with 5 μg (10 μl) anti-RNAPII CTD antibody (8WG16 clone) or 5 μg (5 μl) anti-pSer2-RNAPII (Cat# ab5095), and was incubated for 2 h or overnight, respectively, at 4°C on a rotating platform. After IP, the beads were washed three times with lysis buffer, twice with Wash buffer I [50 mM HEPES, pH 7.5, 500 mM NaCl, 2 mM EDTA, 1% (w/v) Triton X-100, 0.1% (w/v) DOC], twice with Wash buffer II [10 mM Tris–HCl, pH 8.0, 250 mM LiCl, 2 mM EDTA, 0.5% (v/v) Igepal CA-630, 0.5% (w/v) DOC], and once with TE buffer (10 mM Tris–HCl, pH 8.0, 1 mM EDTA). A total of 390 μl or 300 μl of IRN buffer were added to the IP beads or the IN samples, respectively. IP and IN samples were incubated with 10 μl RNAse A (10 mg/ml) at 37°C for 30 min. Then, SDS was added to a final concentration of 0.5% together with 10 μl Proteinase K (10 mg/ml) and the samples were incubated for 1 h at 56°C. Crosslinking was reversed at 65°C overnight. The next day, 1 ng spike-in plasmids (K18) were added in each tube and DNA was isolated by phenol–chloroform–isoamylalcohol extraction. Samples were washed with 70% EtOH, were resuspended in 50 μl ddH_2_O, and stored at −80°C. Samples were diluted 10× and enrichment over IN of the desired sequences was estimated with qPCR, using the iTaq Universal SYBR Green Supermix (Bio-Rad) with the indicated primers (Supplementary Table S3). Data were plotted with GraphPad Prism 9.

### ChIP library preparation and sequencing

Sequencing libraries for ChIP-seq were prepared using NEBNext® Ultra™ II DNA Library Prep with Sample Purification Beads according to manufacturer’s protocol. To account for the smaller amounts of immunoprecipitated materials in chromatin immunoprecipitation (ChIP), adaptor was diluted 10-fold before adaptor ligation. Libraries were amplified with three and seven cycles of PCR for IN and IP samples, respectively. Fragment size and quantification of amplified libraries were analysed on Fragment Analyzer (Agilent) and size selected with QIAseq® Beads if needed before library pooling and sequencing. Libraries were sequenced for 150 cycles in paired-end mode on the Illumina NovaSeq X+ 1.5B platform.

### ChIP-seq data processing

Raw sequencing reads were aligned to *Saccharomyces cerevisiae* R64-1-1 (Ensembl release 115). Because the strains carry the ectopic R-loop-forming ‘mAIRN’ cassette, two derivative references were built by inserting a 2909-bp mAIRN construct into chromosome IV at position 336187 (325-bp/412-bp 5′/3′ homology arms; no genomic sequence deleted) in either of two orientations (‘CoD’ and ‘HO’; modified chrIV = 1534842 bp versus 1531933 bp parental). Each ChIP-seq sample was mapped to the genotype-appropriate reference (CoD- or HO-background genome). Paired-end reads were aligned with Bowtie2 (v2.5; -X 1000 --no-discordant --no-mixed). Properly paired alignments (SAM flag 0×2) were retained with SAMtools. Fragment intervals (outer coordinates of read pairs) were derived with BEDTools (bamToBed -bedpe). Per-base fragment-pileup coverage was computed in R from the imported BED as a GRanges object (rtracklayer::coverage) and exported as BigWig.

### Subsampling and coverage normalization

To normalize for differences in sequencing depth, all IP samples were subsampled to a common read count prior to signal extraction. Aligned reads for each IP sample were loaded as GRanges objects from the pipeline output. The minimum read count across all samples was identified, and each sample was randomly downsampled—without replacement—to that minimum using base::sample. Genome-wide coverage was then computed from the subsampled reads using GenomicRanges::coverage and exported as BigWig files with rtracklayer::export.bw.

For CoD and HO gene analysis, a curated set of 219 CoD and 191 HO open reading frames [15] was mapped to SGD gene identifiers using TxDb.Scerevisiae.UCSC.sacCer3.sgdGene v3.2.2 and org.Sc.sgd.db v3.22.0.

### Stalling index calculation

The stalling index (SI) was calculated from subsampled ChIP-seq BigWig files. Two strand-aware 300 bp windows were defined: a promoter-proximal window spanning the 300 bp upstream of mAIRN TSS and a gene-body-distal window spanning the last 300 bp upstream of the mAIRN TES. Mean coverage within each window was extracted from per-sample BigWig files using rtracklayer. The SI was computed per replicate as follows:


\begin{eqnarray*}
\mathrm{ SI} = {{\log }_2}\left( {\frac{{\mathrm{ sum}\, \mathrm{signal}\left( {\mathrm{star}{{t}_{300\mathrm{ bp}}}} \right) + 1}}{{\mathrm{ sum}\, \mathrm{signal}\left( {\mathrm{ en{{d}}_{300\mathrm{ bp}}}} \right) + 1}}} \right).
\end{eqnarray*}

### RNAPII ChEC assay

Samples and buffers were kept on ice at all times. Frozen fixed cell pellets were washed with 500 μl Buffer A (15 mM Tris–HCl, pH 7.4, 0.2 mM Spermine, 0.5 mM Spermidine, 80 mM KCl, pH 8, 3 mM EDTA, 1× protease and phosphatase inhibitors) and then they were centrifuged at 20 000 × *g* for 1.5 min at 4°C. The supernatant was discarded and cell pellets were resuspended with 500 μl Buffer A. Approximately 500 μl precooled glass beads (1 mm) were added to cover the whole suspension. Cells were disrupted on a VXR basic IKA Vibrax orbital shaker at 2200 rpm at 4°C for 15 min, three times with 10-min breaks on ice in between. The bottom and top of the 1.5 ml tube was pierced using a hot needle and placed into a 15-ml falcon tube. After centrifugation (130 × *g*, 2 min at 4°C), the beads remained in the 1.5-ml tube and the lysate could be collected in the 15-ml tubes. The crude cell lysates were transferred into new 1.5-ml microtubes and centrifuged at 20 000 × *g* for 2 min at 4°C.

The supernatants were discarded and the pellets were resuspended in 500 μl Buffer A. After another centrifugation step (20 000 × g for 2 min at 4°C), supernatants were discarded and cell solutions were resuspended in 350 μl Buffer Ag (Buffer A without EDTA, with the addition of 0.1 mM EGTA) for a total volume of 400 - 450 μl. Samples were incubated for 3 min at 30° at 750 rpm. Then, samples were vortexed vigorously and 80 μl of suspensions were transferred to fresh DNA-low binding tubes containing 100 μl of IRN buffer, to be used as controls (0 min ChEC). 7 μl of 0.1 M CaCl_2_ (2 μl per 100 μl of cell solution, final concentration 2 mM) was added in each tube and the samples were incubated at 30°C with constant shaking (750 rpm) for the activation of MNase digestion. After 10, 30, and 60 min of incubation, 80 μl of the samples were aliquoted into 1.5-ml DNA-low binding tubes containing 100 μl of IRN buffer and mixed to stop the MNase activity.

All samples were incubated with 4 μl of RNase A (10 mg/ml) at 37°C for at least 1 h. Ten microlitres of 10% SDS (final concentration 0.5%) and 4 μl of proteinase K (10 mg/ml) were added, mixed, and samples were incubated for 1 h at 56°C. Crosslinking was reversed by incubation at 65°C overnight. The next day, digested DNA was isolated by phenol–chloroform–isoamylalcohol (25:24:1) extraction. Purified DNA was washed with 70% EtOH and was resuspended in 25 μl ddH_2_O. Twelve microlitres of each sample was transferred to fresh 1.5-ml tubes and were incubated with 2 μl Cutsmart buffer (NEB), 1.5 μl XmaI, and 1.5 μl PmlI restriction enzymes (NEB) each (30 and 15 U, respectively) and 3 μl ddH_2_O (total volume 20 μl) at 37°C overnight, to isolate the mAIRN reporter sequence (∼1.4 kb) for further detection. Next day, restricted samples were mixed with 4 μl purple loading dye 6× (NEB) and were loaded on a 1% agarose gel for the size-separation of the purified DNA and the digested and restricted DNA fragments. The agarose gel containing the separated DNA fragments was incubated with 0.2 N HCl for 20 min at RT. Then, it was rinsed with H_2_O, washed twice with denaturation solution (0.5 M NaOH, 1.5 M NaCl) for 15 min at RT, rinsed once again with ddH_2_O, and washed twice with transfer buffer (1 M NH_4_AcO).

### Southern blot

For the Southern blot transfer assembly, a platform was placed in a tray containing transfer buffer (1 M NH_4_AcO). A wet Whatman paper, free of air bubbles, was placed on the platform. This paper served as a wick to draw the transfer buffer upwards through the gel. The gel was then carefully placed face-down on the wet Whatman paper. A pre-wet nylon membrane was positioned on top of the gel, ensuring no air bubbles were trapped. Three additional Whatman paper sheets were placed on the membrane, followed by a stack of paper towels and a light weight (~0.5 kg). The entire assembly was left undisturbed overnight to allow complete transfer of the DNA fragments. Transfer times varied depending on the fragment size, with fragments up to 15 kb requiring ~18 h. Following transfer, the membrane was UV crosslinked using the Stratalinker autocrosslinking function to covalently bind the DNA fragments, enhancing hybridization signals during subsequent detection steps. The membrane could then be dried and stored at room temperature for further analysis. The DNA probe (294 bp) detecting the mAIRN locus was generated by PCR using primers mAIRN-3-SB-Frw and mAIRN-3-SB-Rev from yeast genomic DNA from yeast strain mAIRN CoD (Y152) as a template (Supplementary Tables S1 and S3). The resulting double-stranded PCR fragment was then used to generate the DNA probe by body labelling using the RadPrime DNA labelling system (Invitrogen) with incorporation of [α−32P] dATP (Hartmann Analytik) according to the instructions of the manufacturer. Membranes were prehybridized for 1 h at hybridization temperature (65°C) with 10–15 ml of hybridization buffer [2× SSC, 0.5 M sodium phosphate buffer, pH 7.2, 7% (v/v) SDS]. Following prehybridization, the buffer was discarded and replaced with 15 ml of fresh prewarmed hybridization buffer. The probe, mixed with salmon sperm DNA (final concentration 100 μg/ml) and boiled for 5 min, was then added to the tube. Hybridization occurred overnight at 60°C–65°C with gentle rotation in a hybridization oven. Blots were washed once with 30 ml of 3x SSC and 0.1% SDS after hybridization. Stringency washes were performed at hybridization temperature with rotation, using three buffers in sequential order: 0.3× SSC with 0.1% SDS, 0.1× SSC with 0.1% SDS, and lastly 0.1× SSC with 1.5% SDS. Each wash step was repeated twice for 15 min. Finally, blots were dried, stored at room temperature, exposed to phosphorimaging screens, and read out using a Typhoon scanner. Blot quantification was performed with ImageJ. Intensity values were normalized to the uncleaved mAIRN sequence (1.4 kb) intensity upon restriction of the ChEC (chromatin endogenous cleavage assay)-treated samples with XmaI and PmlI.

### RNA extraction, cDNA synthesis, and Reverse-Transcription quantitative PCR (RT-qPCR)

Yeast cells were grown to OD_600_ = 1 and 2 ml were harvested and centrifuged for 5 min. For the induction of PP7-mAIRN, cells were incubated with 2% GAL for 2 h. Total RNA was isolated with the RNeasy Kit (QIAGEN) according to the manufacturer’s protocol, using zymolyase digestion (100 U per sample). Purified RNA samples were incubated with DNase for the removal of DNA, according to the manufacturer’s protocol (QIAGEN). Complementary DNA (cDNA) was synthesized with the SuperscriptIII First Strand Synthesis System (Invitrogen) according to the manufacturer’s protocol, using poly-dT primers. Quantification of PP7-mAIRN transcription levels was conducted with quantitative RT-PCR using the iTAQ Universal SYBR Green Supermix (Bio-Rad) according to the manufacturer’s protocol. Reactions were conducted with 0.4 ng of cDNA template, in triplicates for each combination of strain/set of primers. ACT1 gene was used as internal control using previously described primers [42]. The sequences of primers used for RT-qPCR are shown in Supplementary Table S3. Data were plotted with GraphPad Prism 9.

### Spot assays

To assess cell viability, spotting assay was performed. Cell-cycle-restricted expression of RNH1 was performed as previously described [43, 44]. Yeast strains used in this assay were generated on the background of S288C (Supplementary Table S1). Cells were grown overnight in YPD at 25°C and OD_600_ was measured in the morning. Cells were then seeded in 200 μl sterile ddH_2_O into a 96-well plate at a density of OD_600_ = 0.5 and serially diluted four times at a 1:10 ratio. The serial dilutions were then spotted onto YPD agar plates and incubated at either 25°C or 30°C. After 24, 48, and 72 h colorimetric images were taken with the Bio-Rad ChemiDoc Touch.

### Statistical analyses

Statistical analysis of replication time data and single-cell transcription intensities was performed using Monte Carlo resampling with 1 000 000 iterations. DRIP- and ChIP-qPCR data were analysed using a linear mixed-effects model, which accounts for variability between experimental replicates. Pairwise comparisons between conditions were corrected for multiple testing using the multivariate t-distribution method. ChEC data were analysed with student’s *t*-test. RT-qPCR data were analysed with One-Way ANOVA.

## Results

### Yeast replisome progression is inherently robust against mAIRN R-loops in wild-type cells

Investigating the impact of R-loop formation on both replisome progression and transcription constitutes a challenge as it requires the simultaneous tracking of both processes at a specific genomic locus. Previously, we have developed such a system for monitoring transcription–replication coordination by combining locus-specific real-time detection of replisome progression and transcription bursting in single *Saccharomyces cerevisiae* cells by live-cell imaging [36]. This approach is based on tracking replisome progression through a single replicon in real-time [45]. The genomic locus of interest is labelled with tandem repeats of bacterial operator sequences, *lacO* and *tetO*, integrated in a defined and increasing distance from the early-efficient replication origin *ARS413*. These arrays are bound by their cognate repressors conjugated with fluorescent proteins; lacI fused to HaloTag, labelled with SiR-Halo dye (LacI-Halo-SiR) and tetR labelled with tdTomato (tetR-tdTomato) (Fig. 1A). This labelling enables the visualization of the chromosomal locus as two colocalizing fluorescent foci and measurement of *lacO/tetO* array duplication, for the real-time monitoring of replisome progression (Fig. 1B) [36, 45].

**Figure 1.**
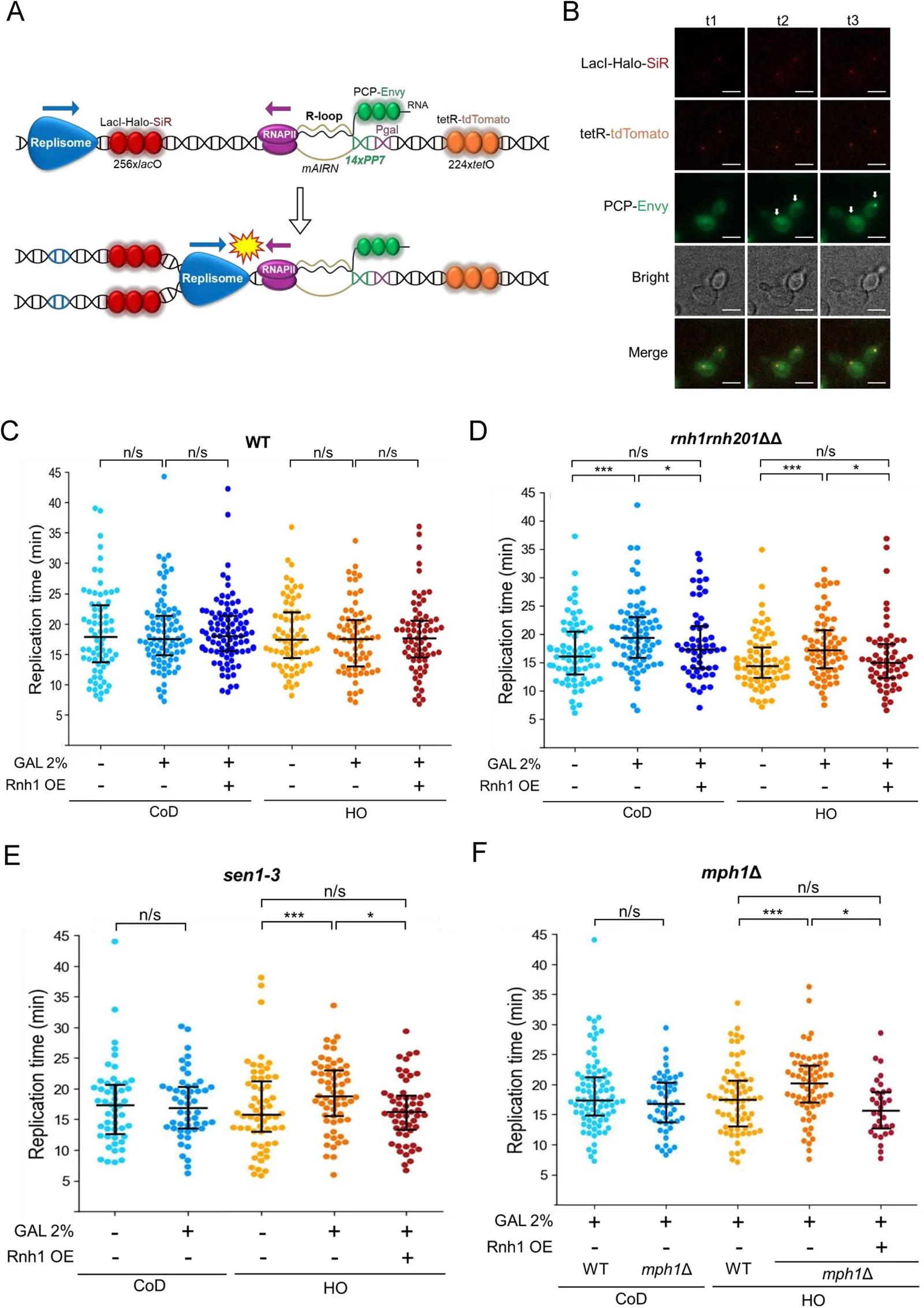
Yeast replisome progression is inherently robust against mAIRN R-loops in wild-type cells. (**A**) Schematic depiction of the live-cell imaging system for measuring replication times through the mAIRN sequence [46] (see text and Tsirkas *et al*. [36] for details). (**B**) Representative cells with LacI-Halo-SiR and tetR-tdTomato-labelled *lacO* and *tetO* arrays, respectively, with PCP-Envy labelled mAIRN transcription bursts located in CoD orientation. PCP-Envy foci, marking transcription bursts, colocalize with the two arrays (white arrows). Different cell cycle stages (t1 = G1-early S, t2 = Mid-late S, t3 = Late S-G2) are shown. Scale bar = 5 μm. (**C**) Replication is not affected by R-loop presence in WT cells. Replication times of mAIRN-expressing cells in CoD and HO, in the absence or presence of 2% GAL, with or without Rnh1 OE. (**D**) Replication is slowed down in *rnh1rnh201ΔΔ* strain upon mAIRN induction in an R-loop-dependent manner. Same as in panel (C), but for *rnh1rnh201*ΔΔ cells. (**E, F**) Replisome progression is slowed down in *sen1-3* and *mph1*Δ cells upon mAIRN induction, only in HO orientation, in an R-loop-dependent manner. Same as panel (C), but for *sen1-3* and *mph1*Δ cells. Median replication times and cell number for each yeast strain and condition are listed in Supplementary Table S6. * = *P < *.05, *** = *P < *.005, **** = *P < *.001 n/s = not significant.

To investigate how R-loop formation interferes with replisome progression on the labelled chromosome arm, we integrated a strong inducible R-loop forming sequence in the path of the replisome (Fig. 1A). Previously, we devised a similar approach to induce a TRC, by integrating the GAL10 gene under the control of its minimal native GAL10 promoter (Pgal) [36] between the two bacterial operator arrays in both CoD and HO orientations. Here, we replaced the GAL10 open reading frame (GAL10orf) with the R-loop-forming sequence of mAIRN [46]. This mAIRN sequence has been previously shown to generate stable and persistent R-loops both *in vitro* and *in vivo*, and it has been utilized for studying the effects of R-loops on replication fork speed, transcription competence, and DNA damage/signalling [20, 47–53]. To measure mAIRN transcription in real-time, we labelled the 5′-end of mAIRN with 14 repeats of the PP7 bacteriophage sequence in cells expressing the bacteriophage PP7 coat protein fused to an optimized GFP (PCP-Envy) [36, 54] (Fig. 1A and B). This system enables high resolution measurement of mAIRN transcription dynamics during replisome passage through the transcribing mAIRN gene.

First, we validated mAIRN R-loop induction in our yeast strains by performing DRIP-qPCR in uninduced or induced conditions using the R-loop-recognizing S9.6 antibody [40]. Galactose induction led to significant R-loop enrichment across the entire mAIRN sequence (m5, m-mid, m3) in both HO and CoD yeast strains. Induction of the GAL regulon also produced a modest, approximately two-fold, increase in R-loop signal at the highly transcribed, R-loop-prone TDH3 gene [8, 15] and at the neighbouring PEX19 locus (Supplementary Fig. S1A), even though neither gene is under Pgal control. This is consistent with previous reports that the shift to galactose as carbon source triggers a genome-wide transcriptional response in yeast extending well beyond the GAL regulon and affecting a substantial fraction of the yeast transcriptome [55]. Since R-loop levels are known to correlate with transcriptional activity [56, 57], this general response likely accounts for the modest increase in DRIP signal observed at TDH3 and PEX19 upon induction. Notably, R-loop levels at PEX19 start from a near-background baseline and even with the increase upon galactose induction remains well below the detected mAIRN and TDH3 R-loop signals (Supplementary Fig. S1A). Importantly, the detected R-loop signal was specific, as the signal was abolished by both *in vivo* RNase H1 overexpression (Rnh1 OE) (Supplementary Fig. S1A and B) and *in vitro Escherichia coli* RnaseH1 treatment (Supplementary Fig. S1C). Finally, galactose induction failed to induce R-loops in the Pgal promoter-driven GAL10orf yeast strain [36], maintaining baseline levels similar to the PEX19 negative control locus (Supplementary Fig. S1D). Together, these results confirm that the detected DRIP signal at the mAIRN locus reflects *bona fide* sequence-specific RNA:DNA hybrids.

Having established the mAIRN locus as a robust model for inducible R-loop formation, we next investigated its impact on replication fork progression. Interestingly, we observed no significant replication slowdown upon mAIRN induction, regardless of whether mAIRN was transcribed in CoD or HO orientation relative to the oncoming replication fork (Fig. 1C and Supplementary Table S6). Consistently, using Rnh1 OE to deplete R-loops had no further effect on replication kinetics (Fig. 1C and Supplementary Table S6). These results indicate that wild-type (WT) replisomes are remarkably robust and can efficiently traverse even a strong R-loop forming sequence during genome duplication.

### Loss of R-loop processing factors reveals orientation-specific barriers

We were intrigued by the robustness of yeast replisome progression through the mAIRN R-loops and hypothesized that, in wild-type cells, the presence of a plethora of R-loop limiting factors, such as RNase H enzymes and RNA:DNA helicases constrains the accumulation of replication-interfering RNA:DNA hybrids [31, 32]. To investigate this in our live-cell imaging system, we initially deleted both catalytic subunits of the two main *S. cerevisiae* RNHs, *rnh1*Δ and *rnh201*Δ (RNH1 and RNH2, respectively) [58]. Replication time of induced RNH mutant cells was significantly longer compared to the uninduced condition, in both CoD and HO orientations (Fig. 1D and Supplementary Table S6). Given that Rnh1 OE in mAIRN-induced cells restored normal replication fork progression (Fig. 1D and Supplementary Table S6), these data indicate that R-loop induction at the mAIRN sequence specifically impedes replication fork progression, likely manifesting as local fork slowing rather than a uniform reduction in replication velocity across the chromosome arm, as previously reported for other R-loop-inducing conditions [59]. These results highlight that RNase H activity is a prerequisite for normal replisome progression through mAIRN R-loops, in agreement with previous research [58].

Beyond enzymatic degradation, we wondered whether the physical unwinding of these structures by dedicated helicases is equally important for fork progression. Thus, we turned our attention to Sen1 and Mph1, two key helicases that provide critical but distinct layers of protection against R-loop-dependent replisome stalling [15, 60–65]. Mph1, the yeast ortholog of the human Fanconi Anemia protein FANCM, canonically functions as a DNA motor protein that resolves branched DNA structures, and is likely also involved in R-loop resolution given its essentiality in the absence of RNHs [63–65]. In contrast to Mph1’s role in DNA structure remodelling, Sen1 is thought to protect the replication fork through its direct association with the replication machinery. To specifically disturb this function, we generated the *sen1-3* mutant (Supplementary Fig. S1E), which is deficient in replisome binding while maintaining its core helicase activity [38]. Live-cell imaging analysis revealed a striking orientation-dependent requirement for these helicases: both *sen1-3* and *mph1*Δ mutations significantly impaired replisome progression during HO encounters while the transcribing mAIRN sequence had no impact on CoD replication (Fig. 1E and F, and Supplementary Table S6). Critically, Rnh1 OE completely suppressed these replication delays, confirming that HO-oriented R-loops constitute a potent physical barrier in the absence of these helicases (Fig. 1E and F, and Supplementary Table S6). These findings demonstrate the exquisite sensitivity of our single-locus system in capturing transient replication delays, therefore providing direct, real-time evidence for the increased hazard of HO R-loops to replisome progression.

### R-loops act as transcriptional suppressors that are relieved by replisome passage

To dissect the temporal relationship between gene expression and R-loop formation before, during, and following replisome progression through the mAIRN gene, we measured transcription kinetics and replication timing using live-cell imaging across hundreds of individual cells as previously described [36] (Fig. 2). Strikingly, mAIRN transcription was extremely low in G1 and early S phase cells, remaining near background levels in both CoD and HO orientations in the majority of cells (Fig. 2A and B, and Supplementary Fig. S2A and B). However, upon replication fork passage through the mAIRN sequence, we observed a robust ∼2-fold (100%) increase in transcriptional intensity (Fig. 2A and B, and Supplementary Fig. S2A and B). This upregulation occurred almost immediately after the replication fork passed the mAIRN gene, suggesting a rapid and efficient activation of gene expression post replication, consistent with previous reports [36, 66]. Notably, this effect was unique to the R-loop prone mAIRN sequence, as the same analysis for the yeast strains expressing the non-R-loop forming PP7-labelled GAL10orf showed only ∼1.2-fold (20%) increase after replisome passage ([36] and Supplementary Fig. S2E), suggesting that the replisome specifically relieves a barrier inherent to R-loop forming loci.

**Figure 2.**
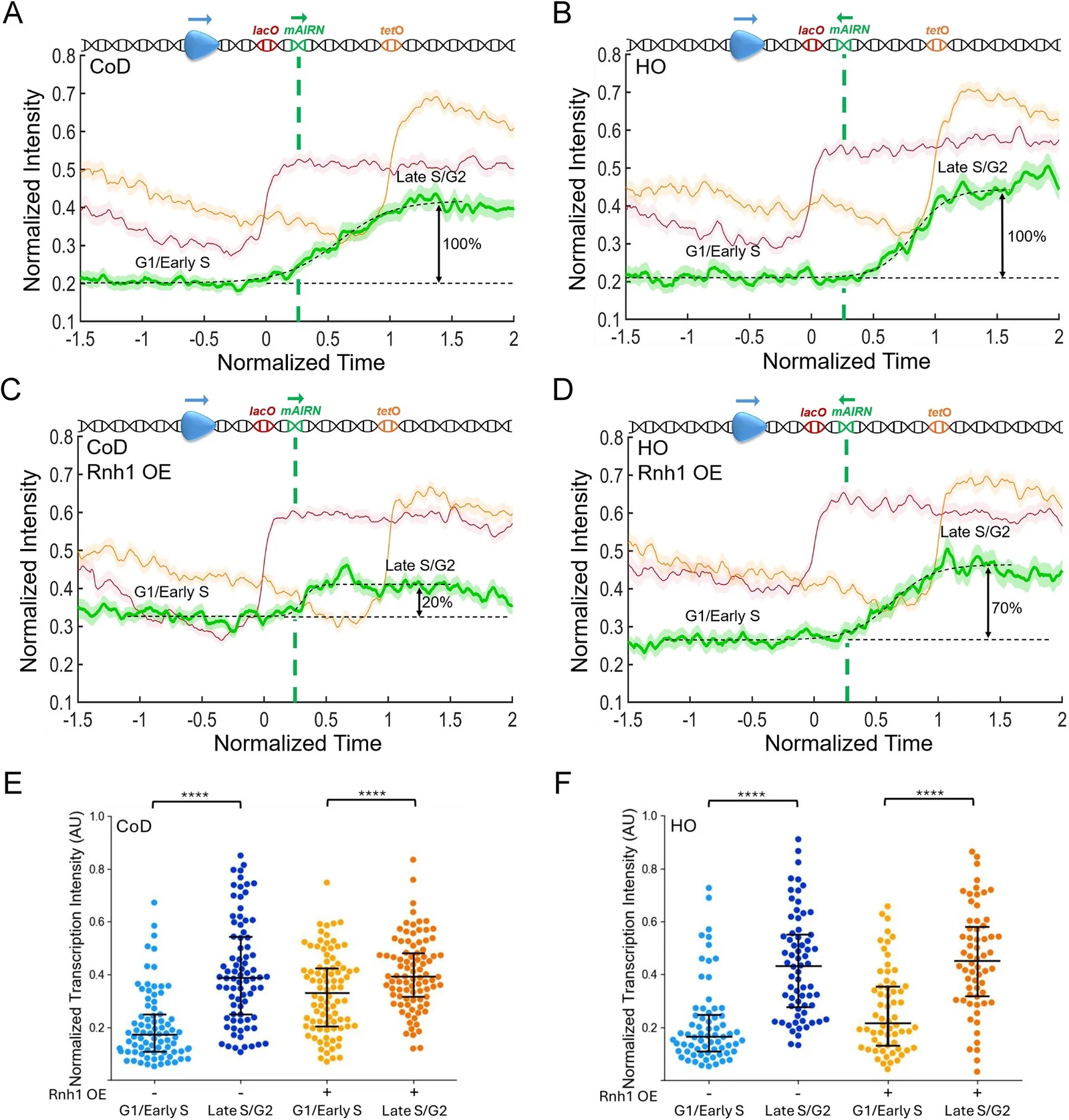
R-loops act as transcriptional suppressors that are relieved by replisome passage. Average normalized fluorescent intensity of the transcription site (PCP-Envy focus, green) and the labelled *lacO* and *tetO* arrays (LacI-Halo-SiR and tetR-tdTomato foci, red and orange, respectively) were plotted over time for cells transcribing mAIRN (2% GAL) in [(**A**) and (**C**)] CoD or [(**B**) and (**D**)] HO orientation relative to the replisome originating from *ARS413*, in [(A) and (B)] WT or [(C) and (D)] Rnh1 OE conditions. The time axis is normalized according to the duplication times of the arrays. The time span 0–1 corresponds to the median replication time per strain under induced conditions (+GAL 2%). Shaded areas represent the standard error of the mean (SEM). A green dashed vertical line represents the timing of mAIRN duplication. Dashed black lines represent a fit of the transcription intensity data to a sigmoidal function. Average SiR-Halo and tdTomato fluorescence signals are shown for highlighting replisome movement through bacterial *lacO* and *tetO* arrays relative to mAIRN transcription and not for comparing replication timing. Top: Scheme showing the relative positions of *ARS413, lacO*, and *tetO* arrays, and mAIRN relative to replisome movement on this locus. Comparison of average single-cell PCP-Envy foci intensities in G1/Early S (before mAIRN replication) or Late S/G2 (post mAIRN replication) phases in mAIRN (**E**) CoD or (**F**) HO yeast strains. Median replication times and cell numbers are listed in Supplementary Table S6. **** = *P < *.001.

We hypothesized that this transcriptional recovery was the direct result of the replisome clearing RNA:DNA hybrids. To test this, we overexpressed Rnh1 in our strains and found that Rnh1 OE significantly elevated basal transcription in G1/early S-phase (Fig. 2C**–** F and Supplementary Fig. S2C and D). This recovery was most pronounced in the CoD orientation (Fig. 2C**–** F and Supplementary Fig. S2C and D), suggesting that while the replisome clears physical obstacles in both orientations, additional orientation-specific constraints may limit HO transcription. Importantly, Rnh1 OE had no impact on the transcription of the PP7-GAL10 control (Tsirkas *et al*. [36] and Supplementary Fig. S2E–G), confirming that the observed transcription repression is strictly R-loop dependent. Although both PP7-GAL10 and PP7-mAIRN constructs are expressed by the identical minimal Pgal, comparison of transcription levels between the two strains via RT-qPCR revealed that mAIRN is expressed 50%–60% less compared to mAIRN upon induction (Supplementary Fig. S2H), suggesting that R-loops are potent transcription impediments, in agreement with our live-cell imaging experiments (Fig. 2 and Supplementary Fig. S2). In addition, the diminished absolute mAIRN transcript levels in *rnh1rnh201ΔΔ* double mutants and their subsequent rescue by Rnh1 OE (Supplementary Fig. S2I) further establish R-loops as potent suppressors of mAIRN expression, which are physically reset by the advancing replisome.

### R-loops impair RNAPII processivity in an orientation-dependent manner

To understand the mechanistic basis of the transcription recovery, we first assessed total RNAPII occupancy using ChIP-qPCR. Consistent with our live-cell imaging experiments, Rnh1 OE significantly increased total RNAPII binding across the mAIRN gene body, whereas no enrichment was observed at the highly expressed ACT1 housekeeping gene (Supplementary Fig. S3A and B). This suggests that R-loops limit the steady-state association of RNAPII with R-loop prone templates, potentially by facilitating the recognition and clearing of stalled complexes, similar to the previously reported mechanisms involving Rat1 or Dcr1 [27, 67].

Next, we examined whether RNAPII binding to mAIRN changes during cell cycle progression. After synchronization in G1 phase, we sampled cells 10 and 50 min post G1 release to test total RNAPII occupancy before and after mAIRN replication, respectively (Supplementary Fig. S3C). As expected, we detected higher RNAPII occupancy on mAIRN gene body in late S compared to early S phase cells, in both CoD and HO mAIRN-expressing yeast strains (Supplementary Fig. S3D). However, despite a major transcriptional upregulation observed in G1/early S phase cells by live cell microscopy (Fig. 2C and D), Rnh1 OE did not majorly affect total RNAPII binding compared to the wild-type profiles (Supplementary Fig. S3D and E). This data suggest that the observed replication-dependent changes in transcription levels prior to replication were not caused exclusively by reduced RNAPII binding on the mAIRN gene but could rather be explained by R-loop-dependent defects in RNAPII elongation.

To address this, we specifically monitored the pool of actively elongating RNAPII molecules by performing ChIP-qPCR for serine-2-phosphorylated RNAPII (pSer2-RNAPII) across five locations spanning the whole mAIRN sequence (Fig. 3A and B). In the CoD orientation, Rnh1 OE led to a robust accumulation of pSer2-RNAPII throughout the gene, consistent with the G1 increase in transcription observed by live-cell imaging (Fig. 2C and E). Interestingly, the strongest enrichment was observed ∼500–600 bp within the central body of the mAIRN sequence (primer pair m-middle), which spans a GC-rich and highly GC-skewed region previously identified as a hotspot for R-loop formation within the mAIRN sequence (Carrasco-Salas *et al*. [68], Fig. 3A, C, and D). In striking contrast, Rnh1 OE in cells expressing mAIRN in HO orientation did not affect the levels of pSer2-RNAPII (Fig. 3B). This result agrees with our live-cell imaging experiments, where Rnh1 OE in mAIRN HO cells showed much weaker recovery of transcription in G1/early S phase cells (Fig. 2D). Together, these results suggest that R-loops are necessary for the efficient processing or removal of stalled RNAPIIs in CoD orientation. In the HO orientation, however, alternative, R-loop-independent mechanisms, such as the heightened torsional stress from the approaching replisome, may dominate RNAPII elongation rate and displacement.

**Figure 3.**
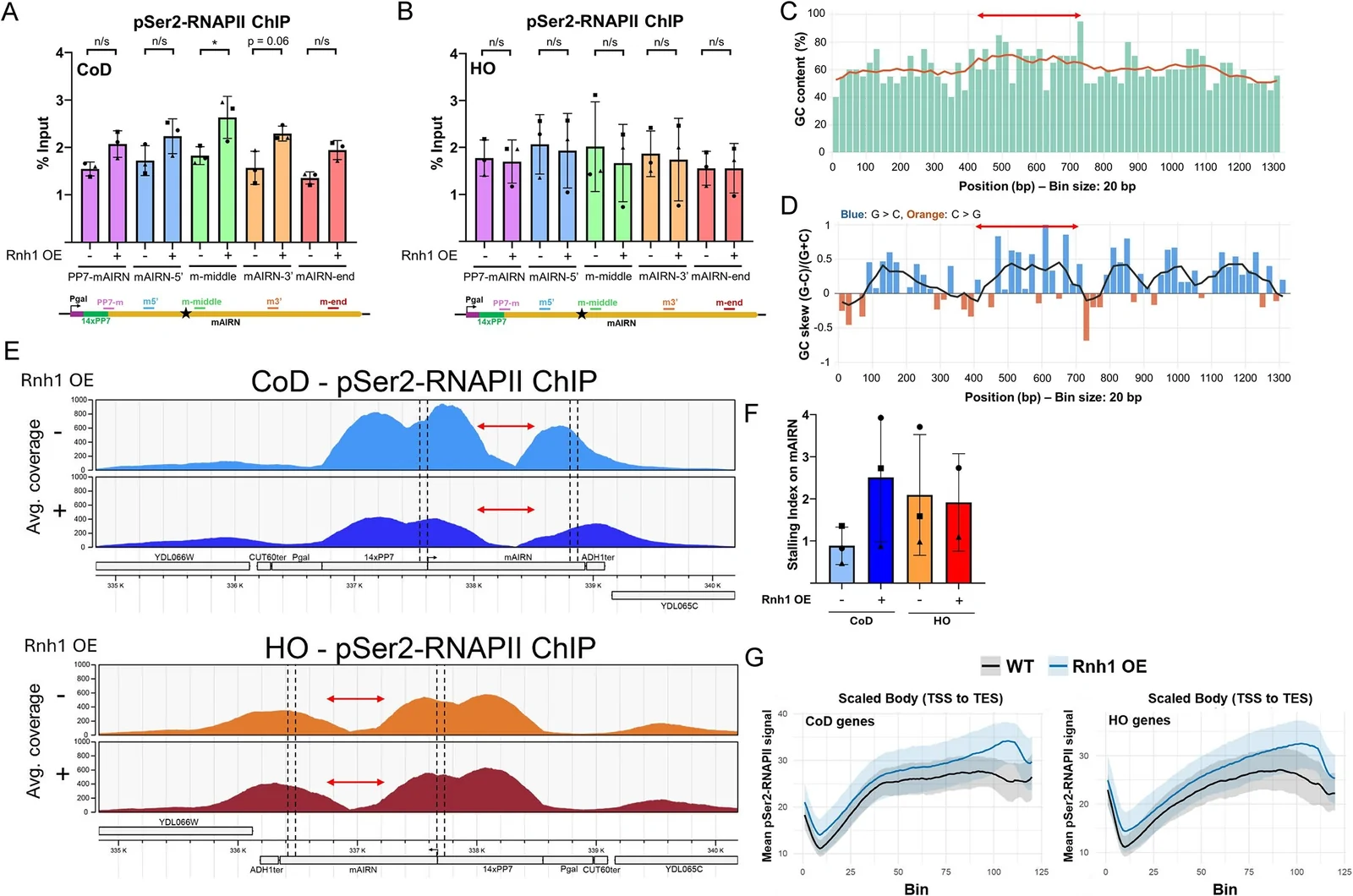
R-loops impair RNAPII processivity in an orientation-dependent manner. pSer2-RNAPII ChIP-qPCR analysis of mAIRN (**A**) CoD- and (**B**) HO-expressing strains. Elevated pSer2-RNAPII occupancy upon Rnh1 OE is only observed in CoD, at 500–600 bp in the middle of the mAIRN sequence. Percent input values were normalized with TDH3. Averages of three biological replicates are shown. Error bars represent ±SD. * = *P < *.05, n/s = not significant. Bottom: Scheme depicting the primer set binding locations on the mAIRN sequence (Supplementary Table S3). The asterisk pinpoints a strong, R-loop forming region on mAIRN (see text) [68]. Graphs depicting the (**C**) GC-content percentage and (**D**) GC-skewness through mAIRN sequence. Bin length = 20 bp. A red and black line, respectively, depict the moving average of the bins (Bins averaged = 8). Horizontal, double-arrowed red lines indicate the central, GC-rich-region of mAIRN. (**E**) Averaged pSer2-RNAPII ChIP-seq reads aligned on the mAIRN reporter sequence elements in CoD or HO strains, with or without Rnh1 OE. Dotted vertical lines highlight the regions used for the calculation of stalling indexes. Horizontal double-arrowed red lines indicate the central, GC-rich-region of mAIRN. (**F**) SI of pSer2-RNAPII for the shown yeast strains, calculated for the windows in the dotted lines in panel (E). (**G**) Mean pSer2-RNAPII signal of 219 CoD and 191 HO genes [15] in WT and Rnh1 OE yeast strains (TSS = transcription start site), TES (transcription end site). Shaded areas represent the SEM.

We further explored these orientation-specific effects using pSer2-RNAPII ChIP-seq analysis. In all conditions, we note that the repetitive, GC-rich central body of mAIRN showed a strong reduction in read depth, which represents likely a PCR bias introduced during standard library preparation for high throughput sequencing (Tilak *et al*. [69] and Fig. 3E, red arrows). To quantify RNAPII processivity without taking into account this central region, we calculated the RNAPII stalling index (SI) defined as the ratio of pSer2-RNAPII ChIP-seq reads in specific bins at the 5′ versus 3′ end of the mAIRN gene (Fig. 3E, grey dashed lines). We found that the SI was higher in HO compared to CoD orientations (Fig. 3F). Notably, Rnh1 OE increased the SI in CoD strains to levels comparable to the HO in the absence of Rnh1 OE, while the latter had no further effect on the HO SI (Fig. 3F). These results suggest that the reduced occupancy of RNAPII observed in CoD strains is driven by R-loop accumulation, as it is sensitive to Rnh1 OE. Furthermore, the lack of an Rnh1-dependent effect in HO strains suggests that stalling in the HO orientation may already be at a functional maximum or is regulated by mechanisms independent of Rnh1-sensitive R-loops (Fig. 3F).

The central region of the mAIRN gene is an R-loop hotspot and likely a site of strong RNAPII pausing, yet this region remained underrepresented in our pSer2-RNAPII ChIP-seq analysis (Fig. 3E, red arrows). To analyse this region at high resolution, we turned to chromatin endogenous cleavage (ChEC) as a complementary readout to visualize individual pausing sites along the mAIRN sequence. To this end, we fused the C-terminus of RPB2, the second-largest RNAPII subunit, with micrococcal nuclease (MNase) (Supplementary Fig. S4A) and performed a ChEC assay followed by Southern blot analysis (Supplementary Fig. S4B). Indeed, ChEC analysis revealed high-intensity cleavage events at the mAIRN centre (500–700 bp) in both orientations, confirming an RNAPII stalling site that coincides with the R-loop hot spot (Supplementary Fig. S4C–F). Crucially, this accumulation was most pronounced in early S phase (pre-replication) and was reduced in late S phase (post-replication) in both HO and CoD orientations (Supplementary Fig. S4C–F), suggesting that replisome passage through the mAIRN sequence resolves or dislodges stalled RNAPII complexes and relieves R-loop-mediated transcriptional pausing as replication progresses.

Finally, to determine whether these locus-specific effects reflect a broader phenomenon, we investigated the impact of Rnh1 OE on global pSer2-RNAPII occupancy. Specifically, we examined pSer2-RNAPII binding levels across a curated set of CoD and HO replication origin-proximal genes previously shown to exhibit 5′-skewed RNAPII occupancy in *sen1-3* mutant cells [15]. Interestingly, Rnh1 OE broadly restored and increased pSer2-RNAPII occupancy across these origin-proximal loci, with a particularly marked accumulation occurring at the 3′ end of the genes (Fig. 3G). This indicates that while removing R-loops relieves transcriptional stalling along the gene body, it promotes elongation down to the 3′ end, consistent with R-loop suppression allowing for efficient transcription elongation.

### The balance between RNAPII binding and R-loop formation prevents harmful TRCs

We were intrigued by the ability of R-loops to obstruct RNAPII occupancy and progression in the context of pre-replicated chromatin, while simultaneously serving as a recognition platform for stalled RNAPII to be efficiently evicted from chromatin [27, 67]. This dual role led us to hypothesize that R-loop formation counter-intuitively might protect genome integrity by limiting RNAPII density, thereby preventing harmful collisions of the replication fork at genes harbouring a high load of RNAPII molecules.

To investigate the interplay between RNAPII binding, R-loop formation, and replication fork stalling, we utilized an *rpb1-1* mutant on the background of the mAIRN strains. This mutant exhibits increased DNA-binding affinity [39] and in our previous work, we found that it leads to replication slowdown upon induced TRCs at the non-R-loop forming *GAL10* gene [36].

To molecularly define the level of RNAPII retention, we performed pSer2-RNAPII ChIP-qPCR in the *rpb1-1* mutant strains with or without Rnh1 OE. In *rpb1-1* CoD cells, R-loop depletion by Rnh1 OE triggered a dramatic increase in RNAPII retention across the entire mAIRN sequence (Fig. 4A). In the HO orientation, Rnh1 OE caused localized RNAPII accumulation specifically in the central region of the mAIRN sequence (Fig. 4B, primer pair m-middle), the exact site where RNAPII stalling was detected in WT cells (Fig. 3A and Supplementary Fig. S4) and where R-loops preferentially accumulate [68]. We further validated these findings using ChEC (Supplementary Fig. S5A and B), which confirmed that Rnh1 OE leads to a significant enrichment of RNAPII occupancy in *rpb1-1* mutant cells at this critical central region (Supplementary Fig. S5C).

**Figure 4.**
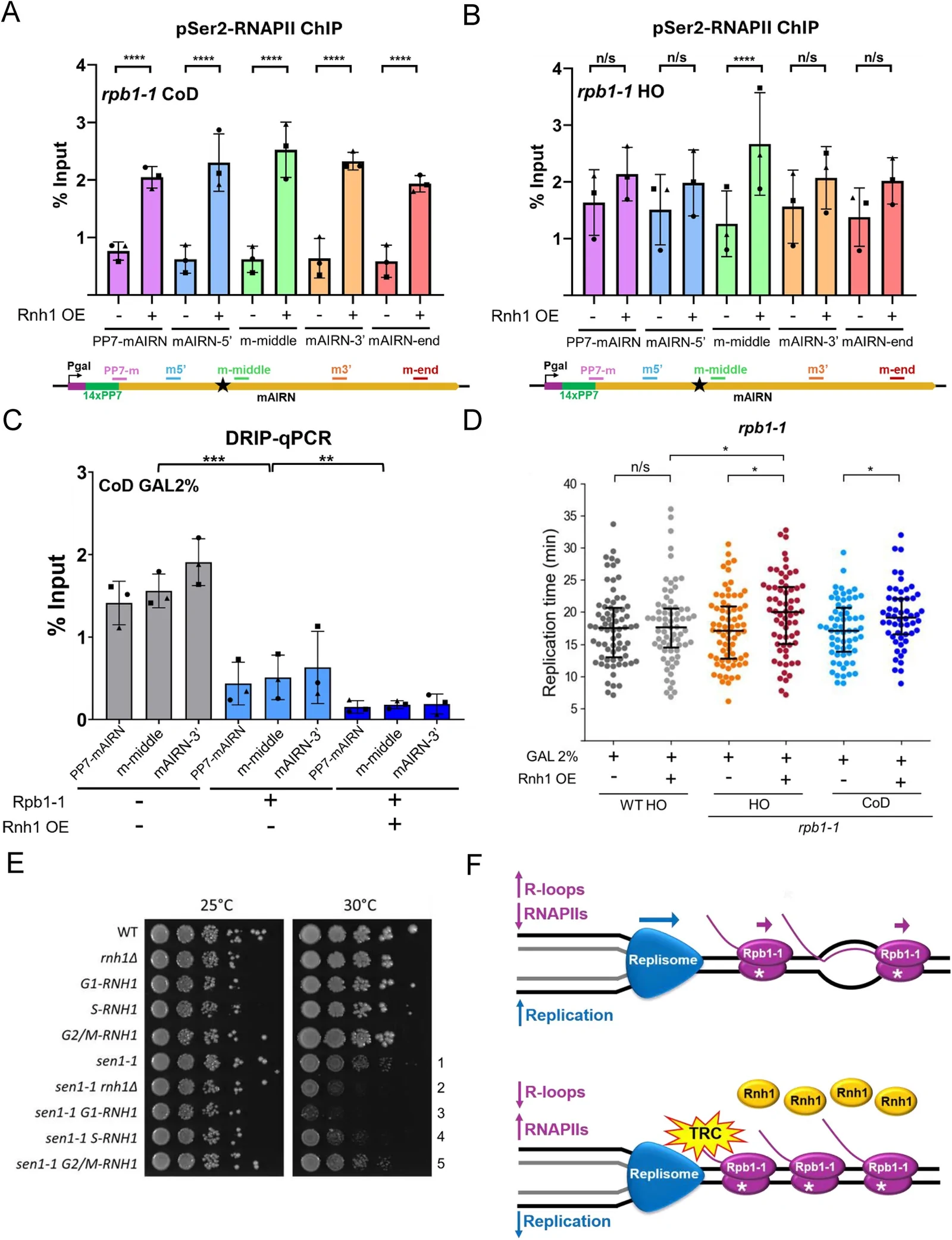
The balance between RNAPII binding and R-loop formation prevents harmful TRCs. R-loop depletion by Rnh1 OE enriches pSer2-RNAPII on mAIRN in (**A**) CoD and (**B**) HO *rpb1-1* cells, especially in the middle of the R-loop reporter sequence. Percent input values were normalized with TDH3. Bottom: Scheme depicting the primer set binding locations on the mAIRN sequence (Supplementary Table S3). (**C**) Competition between RNAPII binding and R-loop formation on mAIRN in *rpb1-1* mutant yeast strain. DRIP-qPCR analysis in *rpb1-1* CoD strain shows reduced R-loop levels compared to WT cells. mAIRN CoD DRIP values (grey) were first shown in Supplementary Fig. S1A and are shown again here for comparison. Averages of three biological replicates are shown. Error bars are ±SD. (**D**) Slowdown of replication in *rpb1-1* cells upon mAIRN induction and Rnh1 OE. WT replication times (grey) are adapted from Fig. 1C and are shown here for comparison. Median replication times and cell number for each yeast strain and condition are listed in Supplementary Table S6. (**E**) R-loop depletion and defective RNAPII removal, especially prior to replisome passage, result to severe growth defect. Spot assays for strains expressing the temperature-sensitive (ts) *sen1-1*, deficient in RNAPII removal, in the absence of rnh1 (*rnh1Δ*) or RNH1 expressed in different cell cycle stages, in permissive or non-permissive temperature. (**F**) Suggested mechanism for the balance between R-loop formation, transcription elongation, and replisome progression in *rpb1-1* cells: R-loops regulate RNAPII binding to mAIRN in *rpb1-1* allowing unperturbed replication. However, R-loops removal leads to excess RNAPIIs binding and replication slowdown. * = *P < *.05, ** = *P < *.01, *** = *P < *.005, **** = *P < *.001, n/s = not significant. Error bars represent ± SD.

Having observed that R-loop-removal in *rpb1-1* mutant cells drives RNAPII accumulation particularly in mAIRN CoD cells, we next assessed baseline R-loop levels in this mutant background. Interestingly, DRIP-qPCR revealed that *rpb1-1* strains exhibit markedly reduced levels of Rnh1 OE-sensitive R-loops across the mAIRN sequence relative to WT (Fig. 4C), suggesting a competition where high-affinity RNAPII binding actively suppresses R-loop formation, whereas intact R-loops restrict excessive RNAPII density along the template.

Next, we evaluated how this delicate equilibrium impacts replisome dynamics by tracking the replication timing of the *rpb1-1* strains using the live-cell microscopy readout (Fig. 1A). Surprisingly, replication progression through the mAIRN gene was unaffected in *rpb1-1* cells, regardless of gene orientation (Fig. 4D and Supplementary Table S6). However, removing the remaining R-loops via Rnh1 OE in *rpb1-1* cells triggered severe replisome slowing in both CoD and HO orientations (Fig. 4D and Supplementary Table S6). These data indicate that R-loop formation at the mAIRN gene normally restricts the high-affinity binding of the *rpb1-1* mutant [39]. When R-loops are cleared, uncontrolled RNAPII ‘piling’ creates a physical barrier that stalls the incoming replisome. Thus, R-loops exert a surprisingly beneficial protective effect by preventing excessive RNAPII density across the mAIRN gene body.

Consistent with the replication patterns observed above, the transcriptional response to R-loop depletion varied significantly by gene orientation. While no major differences were detected in the mAIRN transcription profile of *rpb1-1* cells expressing mAIRN in CoD compared to HO, a slight increase of transcription signal was observed upon Rnh1 OE in *rpb1-1* HO cells, specifically in the early S, before mAIRN sequence is replicated (Supplementary Fig. S5D–F). In contrast, CoD *rpb1-1* cells presented a tremendous increase of mAIRN transcript levels upon Rnh1 OE during the whole duration of Late G1/Early S phase (Supplementary Fig. S5G–I), similar to WT CoD (Fig. 2). Such transcription elevation could stem from the inability of *rpb1-1*-mutant cells to properly displace RNAPIIs downstream of the incoming replisome, eventually resulting in their accumulation and an unavoidable replisome stalling, as detected with our live-cell imaging system (Fig. 4D).

Collectively, these results point towards an unexpected, protective role for R-loops: by restricting the number of RNAPII molecules occupying the DNA template, R-loops prevent the formation of high-density RNAPII ‘trains’. These results suggest that multiple, closely-spaced RNAPII complexes constitute a more severe barrier to the replisome than a single R-loop-associated transcription unit, providing a new perspective on how cells balance transcriptional output with genome stability.

Finally, we wanted to test the functional necessity of this balance between R-loop levels and RNAPII occupancy for genomic stability and cell viability. To this end, we utilized the temperature-sensitive, transcription-termination-deficient *sen1-1* mutant [44] and assessed cell viability when combined with either the absence of RNH1 *(rnh1Δ)*, or with cell-cycle-specific RNH1 expression (G1-, S-, or G2/M-RNH1) [43]. Viability was severely diminished in the *sen1-1 rnh1Δ* (Fig. 4E, lanes 1 and 2), highlighting that when RNAPII removal is defective, the simultaneous persistence of RNAPII complexes and stable R-loops is extremely detrimental [44]. However, while RNH1 expression during S or G2/M phases partially restored growth (Fig. 4E, lanes 4 and 5), a surprising phenotype emerged when RNH1 was expressed exclusively in G1; prior to DNA replication, this condition caused cell viability to drop even more drastically than in the *sen1-1 rnh1Δ* background (Fig. 4E, lane 3). These results, which align with our live-cell imaging data (Fig. 4D), underscore our hypothesis that the complete absence of R-loops, when combined with inefficient transcription termination and RNAPII accumulation, is lethal. Consequently, these findings suggest that R-loops may paradoxically serve to ‘buffer’ or reduce RNAPII queuing, thereby preventing excessive RNAPII accumulation prior to the arrival of the replication fork and averting harmful TRCs that would otherwise lead to replication catastrophe and cell death (Fig. 4F).

## Discussion

Our findings reveal a dynamic crosstalk between replisome progression and transcription, where R-loops act as a primary regulator of this interface. While R-loops have been primarily viewed as drivers of replication stress, DNA damage, and mutagenesis, our real time, single-locus analysis reveals a more complex picture. While the yeast replisome is optimized and well equipped with multiple R-loop processing factors to efficiently bypass R-loops, these structures appear to serve a dual purpose: they repress transcription processivity to prevent the accumulation of multiple RNAPIIs, thereby protecting the replication fork from more severe physical barriers.

### Yeast replisome progression through R-loops is robust but relies on orientation-specific safeguarding factors

Our results show that in WT cells, the replisome is remarkably robust and can efficiently progress through the mAIRN R-loop forming sequence, regardless of orientation (HO or CoD) (Fig. 1C). This finding contrasts sharply with recent reports in human cells, where integration of the same sequence was found to drive significant replication stress and genomic instability [53]. These results highlight the redundant and efficient nature of yeast R-loop processing, which may be an evolutionary adaptation to the high gene density of the yeast genome, where the replisome must constantly navigate through transcription units. However, this robustness appears not to be inherent to the core replisome alone but relies on a specialized and in part redundant ‘toolkit’ of R-loop processing factors. Indeed, only when the catalytic subunits of both RNase H enzymes (*rnh1* and *rnh201)* were deleted, we observed significant replisome slowing at mAIRN R-loops in both HO and CoD orientations (Fig. 1D). In contrast, the specialized roles of Sen1 and Mph1 emerge only during HO encounters (Fig. 1E and F). This orientation-specific dependency aligns closely with recent evidence that harmful R-loop accumulation and its resolution are strictly regulated in a cell cycle-dependent manner [34]. Specifically, Sen1 activity and expression are upregulated during S phase [33], where Sen1 acts to selectively prevent R-loop accumulation and TRCs primarily in the HO orientation compared to CoD orientations [34]. This suggests that HO R-loops present a unique topological or structural challenge during S phase, likely exacerbated by the convergence of replication and transcription machineries and the accumulation of positive supercoiling, which requires dedicated, S-phase-active helicases like Sen1 to prevent fork stalling. Nevertheless, we cannot formally exclude that transient replisome stalling still occurs in WT, particularly in HO orientation, but remains undetected given the time resolution of our live cell imaging methodology (1 min) or the switch in carbon source from RAF to RAF+GAL necessary for mAIRN induction.

### The mechanism(s) of R-loop mediated transcription repression

One of our most striking findings is that mAIRN transcription is suppressed in G1 and early S phase, only to be ‘unlocked’ by the passage of the replication fork (Fig. 2 and Supplementary Fig. S2). This observation aligns with reports that RNA molecules hybridized with the complementary DNA strand act as potent obstacles to RNAPII elongation *in vivo* and *in vitro* [24, 25, 70–75]. We propose that this replication-dependent recovery, which can be partially mimicked by Rnh1 OE-mediated R-loop removal (Fig. 2C and D, and Supplementary Fig. S3), points to an active R-loop removal mechanism coupled to the replication machinery that serves as a global ‘physical reset’, liberating the DNA template from persistent R-loops, and stalled transcriptional complexes that accumulate prior to S phase.

This clearance can be mediated by a multi-layered response: First, the recruitment of R-loop processing factors, such as Rnase H enzymes and Sen1, have been shown to directly interact with replisome components to facilitate progression through transcription–replication encounters [76, 77]. Whether Mph1 also directly interacts with the replisome remains to be studied. Beyond the recruitment of specific factors, this ‘physical reset’ may also involve the direct mechanical dissociation of RNA molecules by the replicative CMG helicase travelling in the front of the leading strand [78] or the FACT complex [79], as well as replication-induced chromatin changes, including histone modifications and nucleosome remodelling, that can inherently inhibit R-loop formation during gene replication [53, 79–82]. Additionally, accumulated topological stress downstream of the replication fork could contribute to the inhibition of R-loop formation. The existence of distinct, orientation-specific clearance mechanisms likely accounts for the differences in transcriptional recovery observed between CoD and HO encounters (Fig. 2 and Supplementary Fig. S2) [20]. Given that in the CoD orientation, transcription occurs on the leading strand template, the CMG helicase may play an important role in R-loop removal enabling high transcription recovery. Ultimately, while replisome progression leads to the full restoration of transcriptional output, the persistence of these inhibitory structures prior to replication hints at a deeper, non-pathological function for R-loops in maintaining genomic stability.

A number of additional mechanisms are likely to contribute to this transcriptional repression, potentially encompassing the coordinated interaction of specialized transcription termination factors and DNA secondary structures. Previous work demonstrated that R-loop-induced RNAPII stalling can trigger Rat1-dependent RNAPII termination [27]. The fact that Rnh1 OE can increase RNAPII occupancy at the mAIRN sequence (Fig. 3A and B, and Supplementary Fig. S3) and can partially recover transcriptional output (Fig. 2 and Supplementary Fig. S2) suggests that R-loop structures may indeed serve as the primary substrate for Rat1-mediated RNAPII termination to actively remove stalled RNAPII molecules to prevent genomic instability. A similar pathway has also recently been described in *Schizosaccharomyces pombe* via Dcr1-mediated RNAPII removal [67]. Thus, it is possible that such R-loop-dependent clearing of RNAPII molecules on mAIRN natively limits its expression in our system. Furthermore, the mAIRN sequence is prone to forming G-quadruplexes (G4s) [52], which may synergize with R-loops to stabilize negative supercoiling of DNA behind actively transcribing RNAPIIs, effectively trapping the polymerase in a low-processivity state [83].

### Orientation-specific constraints on transcription recovery

Interestingly, upon R-loop depletion by Rnh1 OE, transcription recovery was stronger in CoD compared to HO-expressed mAIRN (Fig. 2). This difference was most pronounced at the central midpoint of the mAIRN sequence—the primary hotspot for R-loop formation (Carrasco-Salas *et al*. [68] and Fig. 3A and B). This is also confirmed by pSer2-RNAPII ChIP-seq, showing that RNAPII stalling is inherently more sensitive to R-loop levels in CoD compared to HO (Fig. 3F).

In contrast, the failure of Rnh1 OE to fully rescue transcription in the HO orientation suggests that R-loops are not the sole impediment in this configuration. During HO encounters, the convergence of the replisome and the transcription machinery is expected to generate the accumulation of positive supercoiling between the two complexes. This heightened torsional stress can serve as a ‘polar roadblock’ [84] and result in severe RNAPII stalling, subsequent backtracking or premature dissociation independent of the RNA:DNA hybrid itself. This is consistent with our previous observation that Top1 depletion, which exacerbates topological stress, impairs replisome progression and transcription through non-R-loop forming sequences such as GAL10 [36].

Collectively, these data suggest a model where mAIRN G4/R-loop formation is the primary determinant of RNAPII processivity in CoD. Interestingly, R-loop-mediated RNAPII stalling, specifically in CoD orientation, has been recently connected with the removal of RNAPII from chromatin at actively transcribed genes in higher eukaryotes, either through CFAP20-mediated salvage at promoter-proximal R-loops or CUL3-KCTD10 driving RNAPII ubiquitination at collision sites [21, 22]. These studies highlight a conserved mechanism where orientation-specific, R-loop-stalled complexes can serve as a platform for RNAPII eviction. However, in the HO orientation, the mechanical and topological constraints of the HO collision may dominate, rendering the transcriptional machinery less responsive to enhanced R-loop processing activity such as Rnh1 OE.

### A paradoxical protective role of R-loops to prevent RNAPII piling

The predominant view of R-loops being pathological structures is challenged by our observations in the *rpb1-1* mutant, where R-loop depletion by Rnh1 OE triggers severe replication delays (Fig. 4D). This reveals a critical regulatory trade-off: while persistent R-loops can be a barrier, the pile-up of RNAPII molecules at high density constitutes a significantly greater threat to genome integrity, as it has been shown previously *in vitro* [47]. Our results suggest that by natively impairing RNAPII processivity and facilitating the eviction of stalled complexes, R-loops prevent the formation of high-density RNAPII ‘trains’ that would otherwise become immobile roadblocks. Consequently, we identify two independent causes of replication fork slowdown: (i) the accumulation of persistent R-loops in the absence of R-loop processing factors like RNHs or RNA:DNA helicases (Fig. 1D**–** F) and (ii) the excessive accumulation of RNAPII molecules on the DNA template (Fig. 4D**–** F).

In this context, R-loops may function as a protective ‘buffer’ to prevent excessive RNAPII binding. In the absence of this buffer, high-affinity polymerases such as the *rpb1-1* mutant or inefficiently terminated complexes accumulate into a dense physical barrier that even a robust yeast replisome cannot overcome [27, 67]. Nonetheless, even in WT cells, mAIRN G4/R-loops serve to prevent excessive RNAPII accumulation (Figs 2 and 3). However, because wild-type RNAPII complexes are promptly cleared by the incoming replisome and its associated termination machinery, Rnh1 OE does not compromise replication dynamics in wild-type cells.

This concept is supported by the catastrophic lethality observed when RNH1 is expressed exclusively in G1 in a *sen1-1* background (Fig. 4E). Given that SEN1 expression and function are naturally restricted to S-phase to counteract orientation-specific R-loop barriers [33, 34], removing the R-loop ‘buffer’ in G1 occurs at a time when Sen1-mediated transcription termination is not fully operational. This could lead to uncontrolled RNAPII piling on the DNA template, generating an immobile physical roadblock that subsequently stalls the oncoming replisome during S-phase.

However, the underlying molecular mechanisms governing the observed lethality in spot assays require further examination. Premature Rnh1 activity in G1 might generate uncharacterized genomic substrates or alter hybrid dynamics, thereby allowing residual, improperly processed R-loops to persist into S phase. In a *sen1* mutant background, where S-phase-specific R-loop resolution is already compromised, these persistent hybrids, combined with deregulated transcription complexes, could act synergistically to block cell-cycle progression and impair colony formation. Future high-resolution, cell-cycle-resolved R-loop mapping will be essential to decipher the precise molecular structure of R-loop substrates across distinct cell-cycle stages in these mutant backgrounds.

It has to be noted that the R-loop reporter used in this study, mAIRN, is a sequence foreign to the yeast genome that has been shown to form especially strong and persistent R-loops [20, 53, 68], a property also evident in our own DRIP experiments, where basal mAIRN R-loop levels under non-induced conditions already reached those of the *bona fide* R-loop forming TDH3 gene (Supplementary Fig. S1A). We attribute this elevated baseline to the well-documented leakiness of the Pgal promoter under non-inducing conditions [85]. Notably, this basal signal appears sequence-specific rather than a generic consequence of promoter leakage: GAL10orf, expressed from the identical minimal Pgal promoter and transcribed even more strongly than mAIRN (Supplementary Fig. S2H), fails to accumulate detectable R-loops under either non-induced or induced conditions (Supplementary Fig. S1D), consistent with previous report [86]. We therefore favour an interpretation in which the intrinsic R-loop-forming propensity of the mAIRN sequence, rather than transcriptional output *per se*, underlies both its elevated basal signal and the further, modest increase observed upon galactose induction.

We acknowledge that establishing a strictly causal, quantitative relationship between R-loop accumulation at mAIRN and the replication phenotypes described here remains inherently challenging, since R-loops cannot currently be induced or removed at this locus independently of the genetic and transcriptional perturbations used to reveal them. We note, however, that several convergent lines of evidence support our model proposed in this study: the RNase H- and helicase-sensitivity of the replication delay (Fig. 1D**–** F), its dependence on transgene orientation relative to the oncoming fork, and the live-cell imaging and functional data linking R-loop loss to aberrant RNAPII accumulation and replisome stalling (Fig. 4). Together, these observations support a model in which stable mAIRN R-loops actively limit harmful RNAPII pile-up.

Furthermore, mAIRN is induced only 2 h prior to the assays used in this study, in an effort to elevate the severity of mAIRN-dependent TRC events. Expanding our observations genome-wide presents additional limitations, since yeast replication and transcription machineries have likely co-evolved with endogenous R-loop-forming sequences over millions of years, developing dedicated mechanisms to circumvent such obstacles and adjust transcriptional output accordingly. As such, the mAIRN sequence is used here as an extreme case to trigger R-loop dependent TRCs that may otherwise remain buffered at native loci, and the present results should be interpreted with appropriate caution when extrapolating to genome-wide effects of R-loops on replication and RNAPII occupancy.

More broadly, given that R-loops are enriched at highly-expressed genes in all domains of life [56, 57], our findings suggest that their ability to regulate RNAPII occupancy represents a conserved beneficial mechanism to prevent harmful TRCs and maintain genome stability. This concept is increasingly supported by studies in mammalian systems, where R-loops serve as crucial structures for controlling RNAPII density at both promoters and terminators. For example at gene promoters, R-loop attenuation and RNAPII traffic control have been shown to be regulated by the SOSS-INTAC complex, where SSB1-mediated recognition of single-stranded DNA triggers promoter-proximal RNAPII termination [87]. At the 3′ end of genes, R-loops formed at G-rich terminator regions are required for proper RNAPII pausing and termination, a process facilitated by R-loop induced antisense transcription that directs DICER/Ago-dependent H3K9me2 deposition and HP1ɣ recruitment [88]. Moreover, these terminator R-loops serve as dedicated platforms to recruit essential termination factors and RNA-modifying machinery. For instance, the RNA helicase DDX21 binds terminator R-loops to recruit METTL3 for m6A-deposition on nascent RNA, promoting XRN2-mediated termination [89]. Similarly, R-loops at termination sites are resolved via the POLR2A R1810me2–SMN pathway, which recruits neurodegenerative disease-associated RNA-binding proteins like TDP-43 and FUS to clear stalled complexes [90]. Crucially, when these regulatory networks or RNAPII removal mechanisms fail, uncontrolled RNAPII accumulation and persistent R-loops trigger widespread TRCs, DNA damage, and genome instability, underlying hallmark traits of cancer and neurodegenerative diseases. Together, these parallels demonstrate that the dual function of R-loops, acting both as transient obstacles and as necessary regulatory buffers for RNAPII traffic, is a universal principle governing genome maintenance from yeast to humans.

## Supplementary Material

gkag1139_Supplemental_File

## Data Availability

The code used for the analysis of live-cell imaging experiments is available online (Supplementary Table S5). pSer2-RNAPII sequencing data have been deposited to NCBI Gene Expression Omnibus under the accession number GSE328746. Any additional information required to re-analyse the data reported in this paper is available from the lead contact upon request.
