# Supplementary material for: The balance between R-loops and RNAPII occupancy determines the outcome of transcription–replication conflicts
Source: Nucleic Acids Res. 2026 Nov 11;54(21):gkag1139. doi: 10.1093/nar/gkag1139 (PMC13752515; doi:10.1093/nar/gkag1139)
Supplement: Supplementary file 1 [file gkag1139_supplemental_file.pdf]

SUPPLEMENTARY INFORMATION

Supplementary Figures

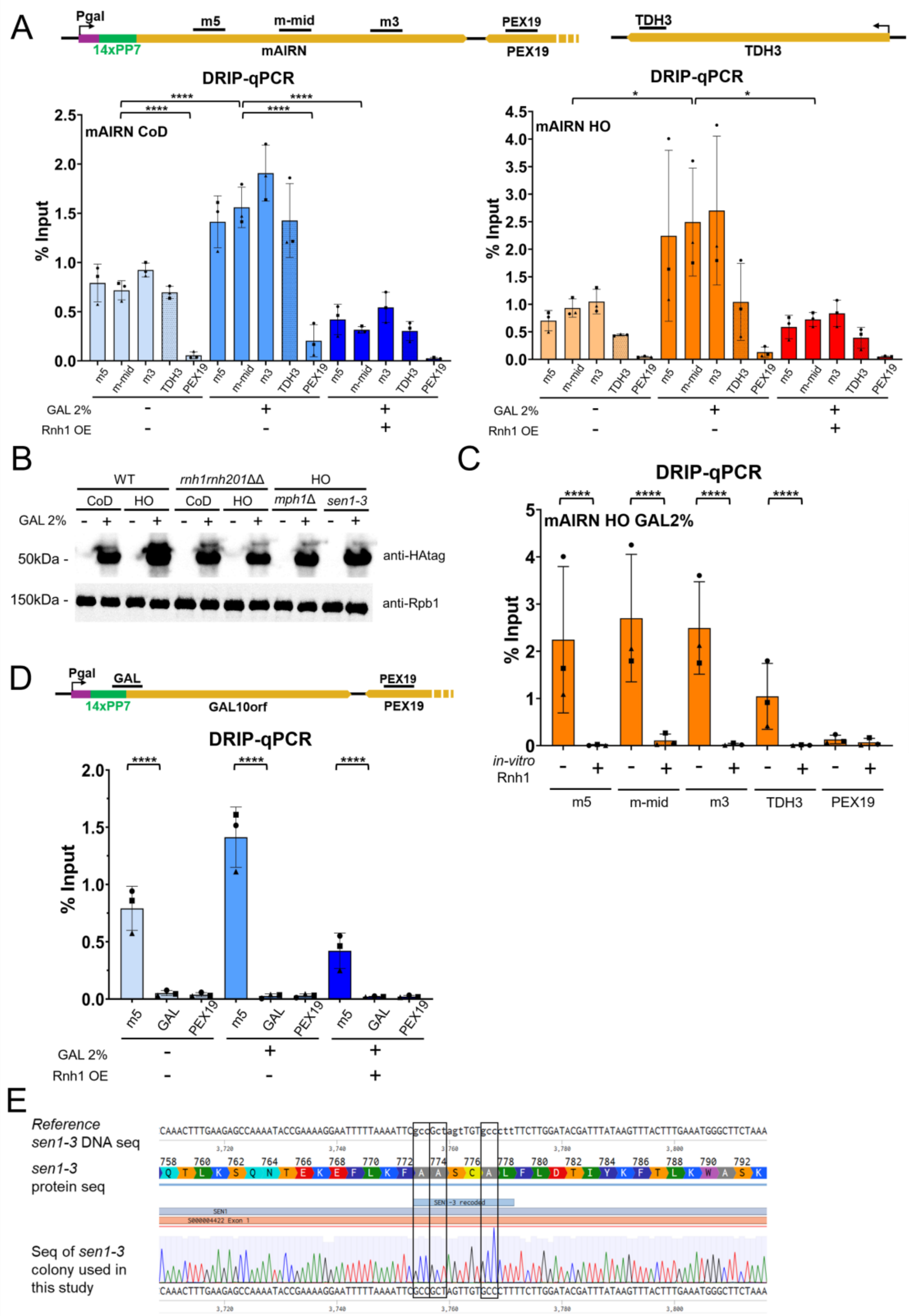

Figure S1. Establishment of yeast strains expressing mAIRN, with or without Rnh1 OE, and the yeast mutant *sen1-3*. (**A**) DRIP-qPCR shows R-loop generation on mAIRN upon its induction with 2% GAL, and R-loop depletion after Rnh1 OE, for both mAIRN CoD (left) and HO (right) yeast strains. R-loops were also detected on the R-loop forming TDH3 gene, but not at a gene downstream of mAIRN integration (PEX19). Top: Schemes depicting the relative location of the amplicons of the primer sets used in DRIP-qPCR experiments and the relative position of PEX19, located donwstream of the integrated Pgal-14xPP7-mAIRN cassette (CoD orientation is shown). Primer sets: m5 = mAIRN5', m-mid = mAIRN-middle, m3 = mAIRN3' (Table S3). (**B**) Representative Western Blot for the detection of Pgal-RNH1-3HA induction, in the absence (-) or presence (+) of galactose in all investigated strains. Anti-Rpb1 antibody is used as loading control. (**C**) *in-vitro* Rnh1 treatment of DRIP samples results to loss of DRIP signal. DRIP-qPCR results of mAIRN HO yeast strain in the presence of GAL2% were initially shown in Fig. S1A and are shown again here for comparison. (**D**) GAL10orf sequence integrated downstream of *ARS*413 locus in CoD orientation [1] showed no R-loop enrichment, similarly to the downstream R-loop negative PEX19 gene. Top: Scheme depicting the relative location of the amplicons of the primer sets used in DRIP-qPCR experiments and the relative position of PEX19, located downstream of the integrated Pgal-14xPP7-GAL10orf cassette. Primer set: PP7-GAL10 (Table S3) DRIP-qPCR results of mAIRN CoD yeast strain (m5 primer set) were initially shown in Fig. S1A and are shown here again for comparison. (**E**) Alignment of Sanger sequencing results of the Y161 yeast strain with the reference *sen1-3* sequence, showing the successful generation of the triple point mutant (W773A, E774A, W777A). Error bars represent ±SD. * = $p < 0.05$, **** = $p < 0.001$.

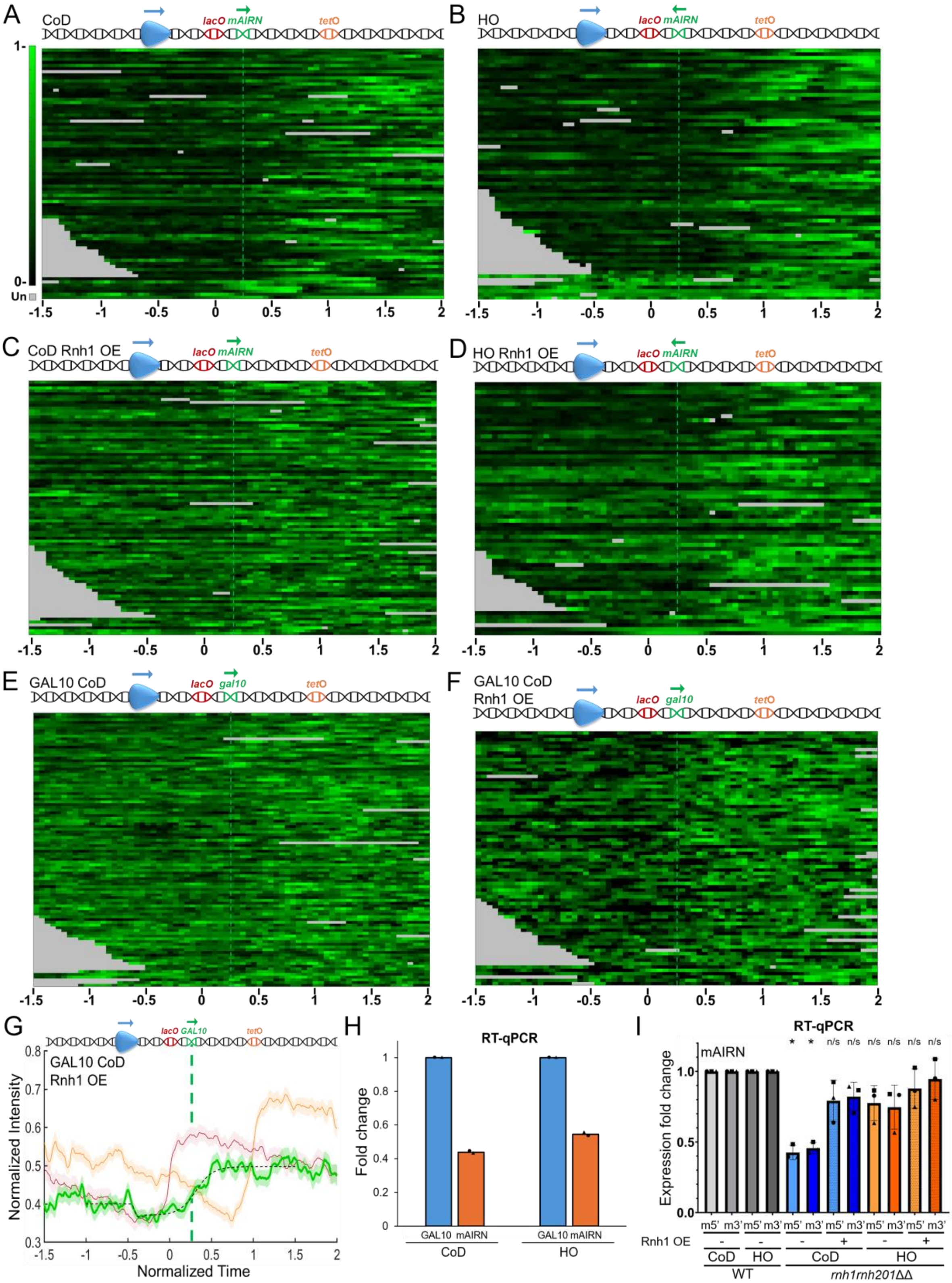
A
CoD
lacO mAIRN
tetO
1-
0-
Un
B
HO
lacO mAIRN
tetO
C
CoD Rnh1 OE
lacO mAIRN
tetO
D
HO Rnh1 OE
lacO mAIRN
tetO
E
GAL10 CoD
lacO gal10
tetO
F
GAL10 CoD
Rnh1 OE
lacO gal10
tetO
G
lacO GAL10
tetO
GAL10 CoD
Rnh1 OE
Normalized Intensity
Normalized Time
H
RT-qPCR
Fold change
GAL10 mAIRN
GAL10 mAIRN
CoD
HO
I
RT-qPCR
mAIRN
Expression fold change
m5' m3' m5' m3' m5' m3' m5' m3' m5' m3' m5' m3' m5' m3'
Rnh1 OE
CoD HO CoD HO
WT
rnh1rnh201ΔΔ

Figure S2. Live-cell imaging of mAIRN nascent transcripts in single *Saccharomyces cerevisiae* cells. (**A-D**) Kymographs depicting PCP-Envy foci intensity through time for cells transcribing mAIRN (2% GAL) (**A-C**) CoD and (**B-D**) HO yeast strains, in (**A-B**) WT or (**C-D**) Rnh1 OE yeast strains. The colour of each bin represents the strength of the PCP-Envy foci normalized fluorescent intensity in single cells (horizontal lines), with the greener the bin colour, the stronger the intensity. The time axis is normalized according to the duplication times of the arrays (see also Fig. 2). Grey bins indicate timepoints that the PCP-Envy foci could not be identified (Un). Top: Scheme showing the relative positions of *ARS413*, *lacO and tetO* arrays, and mAIRN relative to replisome movement on this locus. (**E-F**) Same as (A-B), but for a yeast strain expressing the Pgal-14xPP7-GAL10 cassette in CoD orientation between the *lacO* and *tetO* arrays in (**E**) WT or (**F**) Rnh1 OE cells. Median replication time and cell number are listed in **Table S6**. See also [1]. (**G**) Same as in **Fig. 2C&D,** but for a yeast strain integrated with the Pgal-14xPP7-GAL10 cassette in CoD orientation between the *lacO and tetO* arrays. (**H**) Comparison of GAL10 and mAIRN expression levels using RT-qPCR in the respective yeast strains with the mAIRN Frw/Rev primer set (Table S3). The average of two biological replicates is shown. (**I**) RT-qPCR analysis of mAIRN expression levels in induced WT or *rnh1rnh201*ΔΔ cells, with or without Rnh1 OE, with the mAIRN5' and mAIRN3' primer sets (Table S3). The average of three biological replicates is shown. Error bars represent ±SD. * = $p < 0.05$, n/s = not significant.

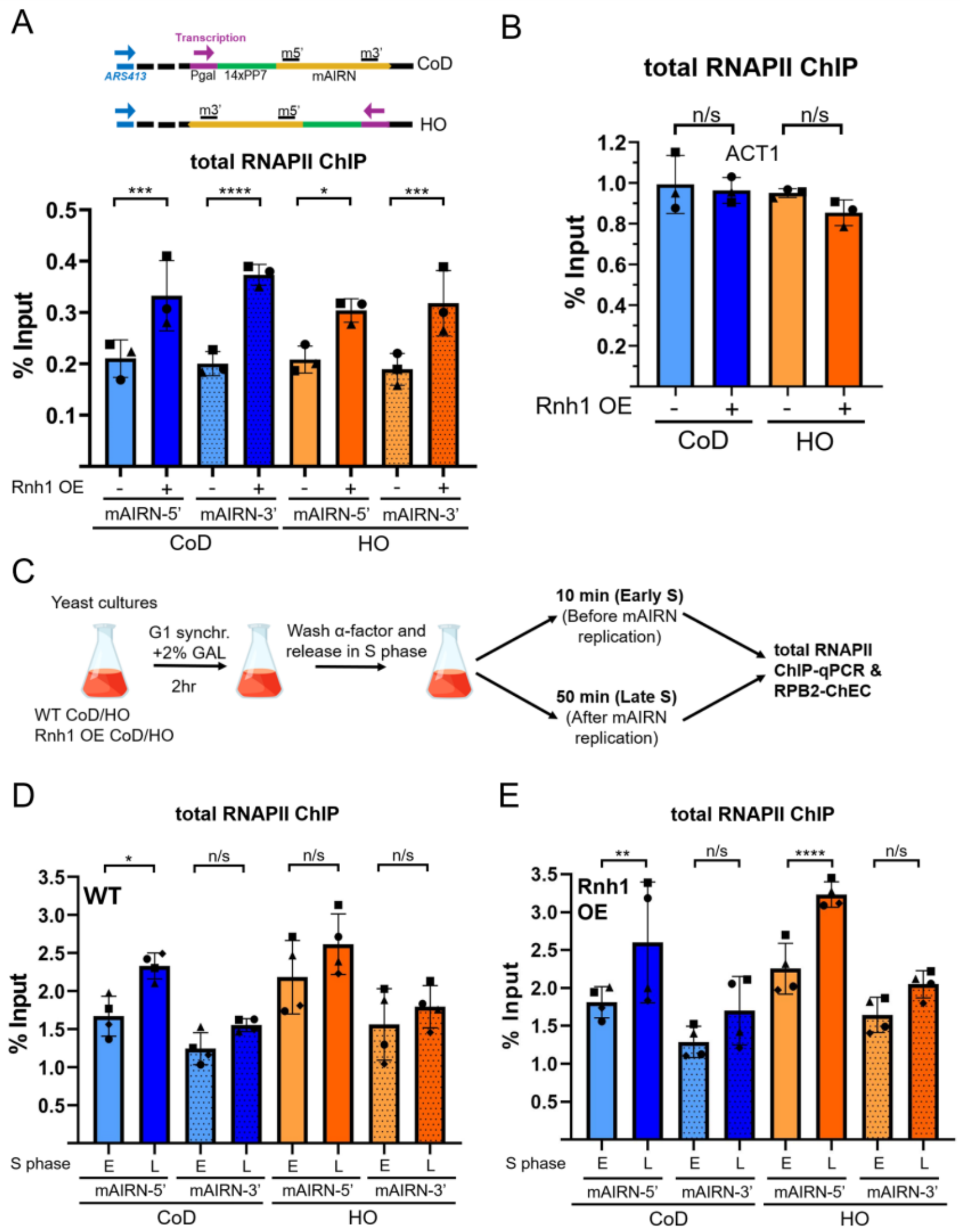

Figure S3. ChIP-qPCR analysis of total RNAPII occupancy on mAIRN. (**A**) Total RNAPII ChIP-qPCR analysis of mAIRN CoD- and HO-expressing strains shows elevated total RNAPII occupancy upon Rnh1 OE. Bottom, scheme of the tested primer sets binding on mAIRN sequence. (**B**) Same analysis as in (A) shows no total RNAPII occupancy changes on ACT1 gene between WT and Rnh1 OE cells. % Input values were normalized with TDH3. Averages of three biological replicates are shown. Error bars represent ±SD. (**C**) Experimental strategy to investigate how replisome progression affects total RNAPII occupancy and activity on mAIRN. (**D-E**) Total RNAPII ChIP-qPCR analysis of (**D**) WT and (**E**) Rnh1 OE CoD and HO strains using the two primer sets used in (A) shows elevated total RNAPII occupancy in Late S phase (L) timepoint (post-mAIRN replication) compared to Early S phase (E) timepoint (pre-mAIRN replication). % Input values were normalized with ACT1. Averages of four biological replicates are shown. Error bars represent ±SD. * = p < 0.05, ** = p < 0.01, *** = p < 0.005, **** = p < 0.001, n/s = not significant.

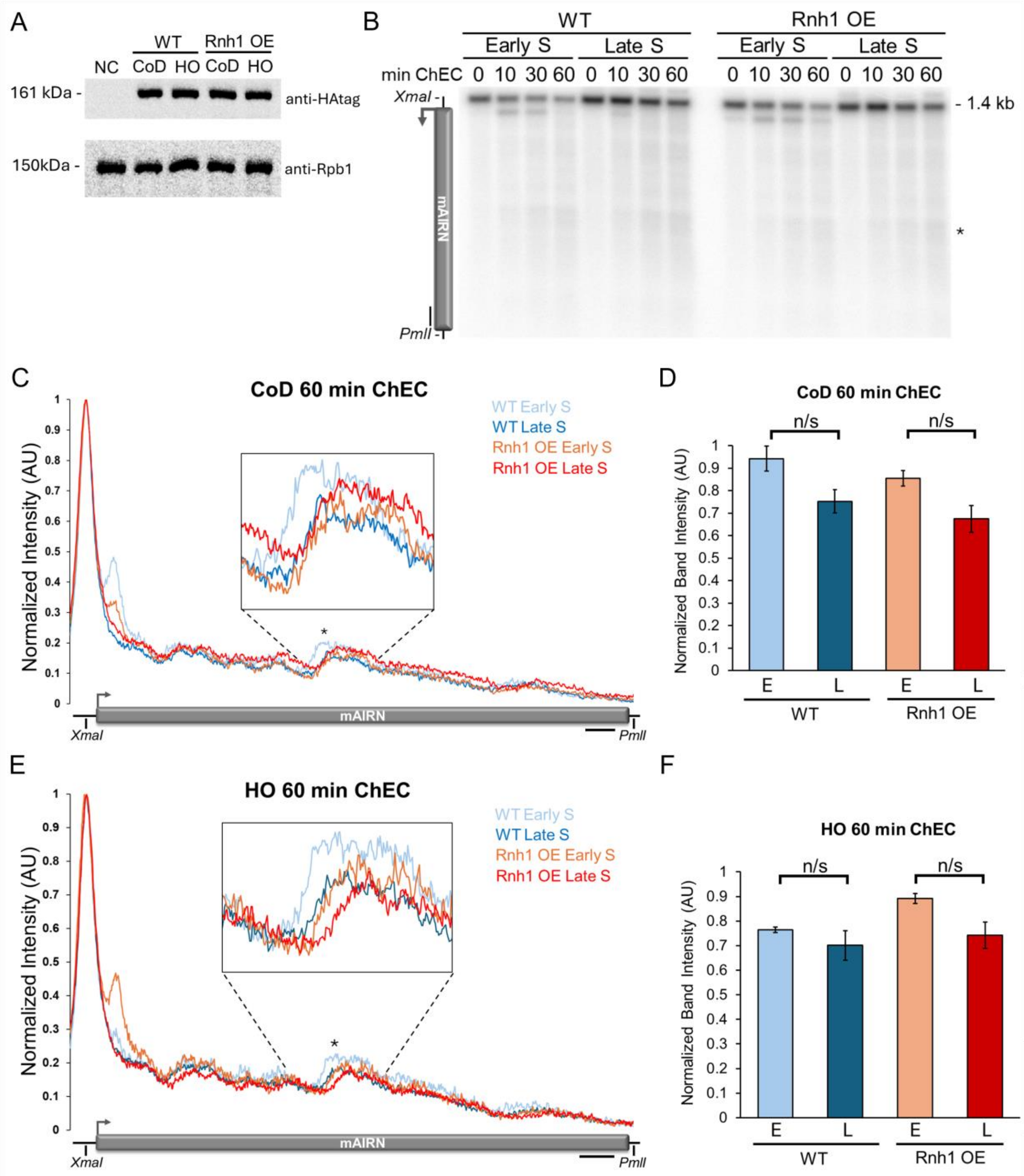
A
NC
WT
CoD
HO
Rnh1 OE
CoD
HO
161 kDa -
anti-HAtag
150kDa -
anti-Rpb1
B
WT
Early S
Late S
Rnh1 OE
Early S
Late S
min ChEC
0 10 30 60 0 10 30 60 0 10 30 60 0 10 30 60
XmaI
mAIRN
PmlI
- 1.4 kb
*
C
CoD 60 min ChEC
WT Early S
WT Late S
Rnh1 OE Early S
Rnh1 OE Late S
Normalized Intensity (AU)
XmaI
mAIRN
PmlI
D
CoD 60 min ChEC
n/s
n/s
Normalized Band Intensity (AU)
E
L
E
L
WT
Rnh1 OE
E
HO 60 min ChEC
WT Early S
WT Late S
Rnh1 OE Early S
Rnh1 OE Late S
Normalized Intensity (AU)
XmaI
mAIRN
PmlI
F
HO 60 min ChEC
n/s
n/s
Normalized Band Intensity (AU)
E
L
E
L
WT
Rnh1 OE

Figure S4: ChEC analysis identifies strong RNAPII stalling sites on mAIRN. (**A**) WB analysis of mAIRN CoD and HO, WT or Rnh1 OE cells, expressing RPB2 fused with MNase and labeled with 3xHA compared to untagged control cells (NC). Anti-Rpb1 antibody was used as a loading control. (**B**) Representative Southern Blot membrane of ChEC samples (0, 10, 30, 60 min) for a strain expressing mAIRN in HO orientation, sampled at Early S or Late S phase, as depicted in Fig. S3C. The asterisk highlights a strong RPB2-stalling event approximately 500-600 bp downstream of mAIRN start. One of two biological replicates is shown. Left: Scheme of mAIRN sequence with marked restriction sites and probe location aligned with the Southern Blot analysis. (**C**) Relative Rpb2-MNase occupancy through mAIRN in CoD orientation yeast strains, both in WT (light and dark blue) and Rnh1 OE (orange and red) cells, sampled as in Fig. S3C (Early or Late S phase), as measured with ChEC-Southern blot upon 60 min MNase activation. Magnification of one of these regions is presented. One of two biological replicates is shown. Bottom: Scheme of mAIRN sequence aligned with the relative Rpb2 occupancy quantification plot. (**D**) Relative quantification of the band corresponding to the strong Rpb2 stalling site (asterisk) in (B) and (C). Averages of two replicates are shown. Error bars are ±SD. n/s = not significant. (**E-F**) As in (**C**) and (**D**), but for HO orientation yeast strains.

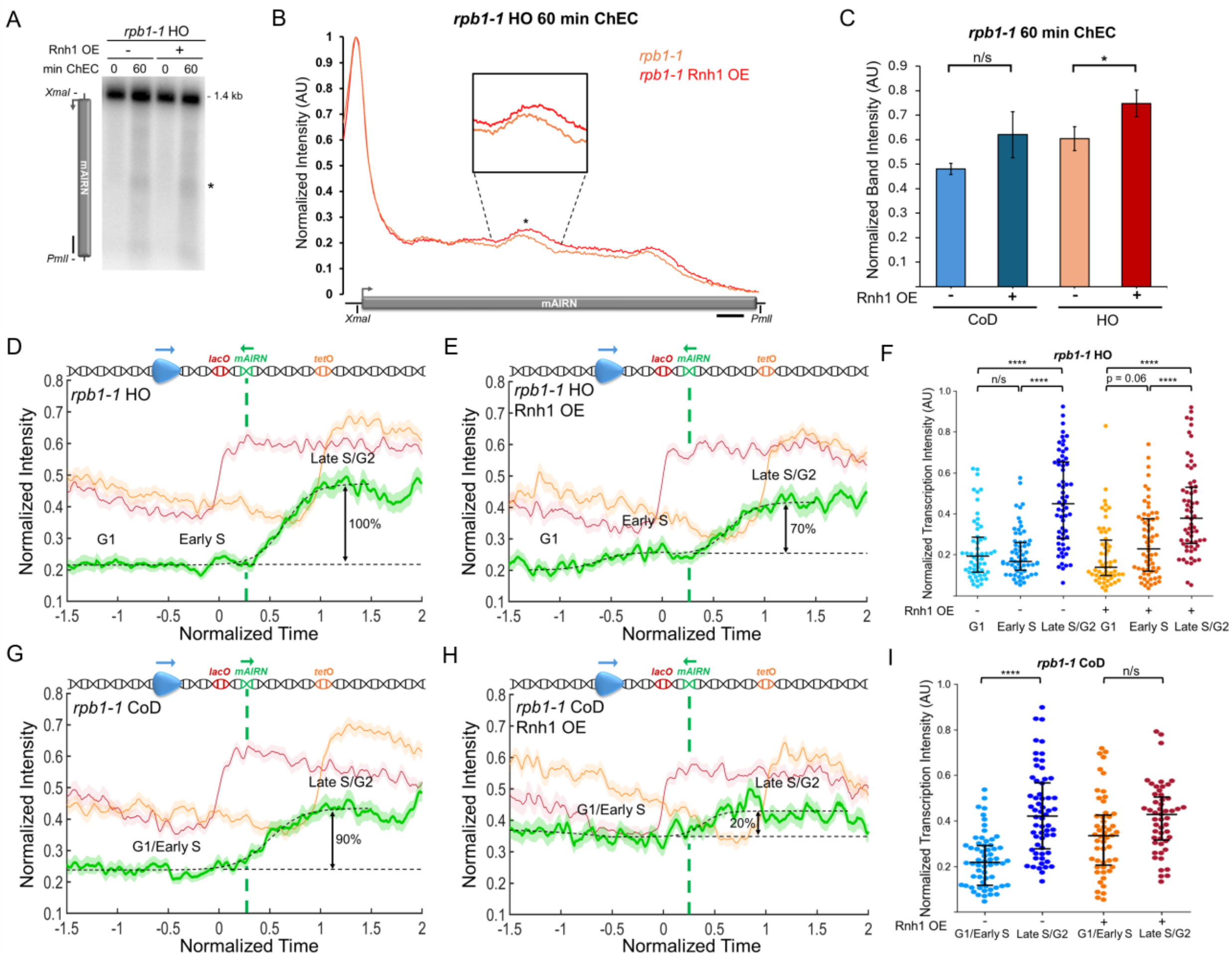

A
rpb1-1 HO
Rnh1 OE - +
min ChEC 0 60 0 60
XmaI
mAIRN
PmlI
1.4 kb
*
B
rpb1-1 HO 60 min ChEC
rpb1-1
rpb1-1 Rnh1 OE
Normalized Intensity (AU)
mAIRN
XmaI
PmlI
C
rpb1-1 60 min ChEC
Normalized Band Intensity (AU)
n/s
*
Rnh1 OE
CoD
HO
D
lacO mAIRN tetO
rpb1-1 HO
Normalized Intensity
Normalized Time
G1
Early S
Late S/G2
100%
E
rpb1-1 HO
Rnh1 OE
G1
Early S
Late S/G2
70%
F
rpb1-1 HO
Normalized Transcription Intensity (AU)
n/s
****
p = 0.06
Rnh1 OE
G1 Early S Late S/G2 G1 Early S Late S/G2
G
rpb1-1 CoD
G1/Early S
Late S/G2
90%
H
rpb1-1 CoD
Rnh1 OE
G1/Early S
Late S/G2
20%
I
rpb1-1 CoD
n/s
****
G1/Early S Late S/G2 G1/Early S Late S/G2

Figure S5. RNAPII binding profile and mAIRN transcription analysis in *rpb1-1* yeast cells. (**A**-**C**) ChEC analysis of Rpb2 occupancy throughout mAIRN in *rpb1-1* yeast cells shows Rpb2 enrichment upon R-loop depletion with Rnh1 OE. (**A**) Representative Southern Blot membrane of ChEC samples (0 and 60 min timepoints). (**B**) Relative Rpb2 occupancy through mAIRN in HO *rpb1-1* yeast strains, without (orange) and with (red) Rnh1 OE cells, as measured with ChEC-Southern blot upon 60 min MNase digestion. The asterisk pinpoints a strong Rpb2-stalling event approximately 500-600 bp downstream of mAIRN start. One of three biological replicates is shown. Bottom: Scheme of mAIRN sequence aligned with the relative RNAPII occupancy quantification plot. (**C**) ChEC analysis of Rpb2 shows increased binding of RNAPII in *rpb1-1* cells upon Rnh1 OE. Relative quantification of the distinct ChEC bands marked with an asterisk (A-B) after 60 min ChEC. Averages of triplicate experiments are shown. (**D**-**E**) Average normalized fluorescent intensity of the transcription site (PCP-Envy focus, green) and the labelled *lacO* and *tetO* arrays (lacI-Halo-SiR and tetR-tdTomaro foci, red and orange respectively) were plotted over time for cells transcribing mAIRN (Induced) in HO *rpb1-1* yeast cells (**D**) without or (**E**) with Rnh1 OE. The time axis is normalized according to the duplication times of the arrays. Shaded areas represent SEM. A green dashed vertical line represents mAIRN duplication time. Dashed black lines represent a fit of the transcription intensity data to a sigmoidal function. Top: Scheme showing the relative positions of *ARS413*, *lacO* and *tetO* arrays, and mAIRN relative to replisome movement on this locus. (**F**) Comparison of average single-cell PCP-Envy foci intensities of WT or Rnh1 OE *rpb1-1* HO yeast cells in G1/Early S and Late S/G2 phase, as shown in (D-E). (**G**-**I**) As in (D-F), but for mAIRN CoD *rpb1-1* cells. * = $p < 0.05$, **** = $p < 0.001$, n/s = not significant.

Table S1: Yeast and bacterial strains used in this study

Identifier	Strain	Genotype	Source
Y152	mAIRN CoD	*Saccharomyces cerevisiae* W303; MATa; leu2-3,112 trp1-1; can1-100; ura3-1; ade2-1; *chrIV*:332961::*lacO*x256-TRP; *chrIV*:352558::*tetO*x224-LEU; *ade1*Δ::tetR-tdTomato+PCP-Envy; *pdr5*Δ::lacI-HaloTag; *chrIV*:336186::CUT60ter-Pgal-PP7x14-mAIRN-ADH1ter transcribed in **CoD** relative to the replisome moving from ARS413	This study
Y153	mAIRN HO	Same as Y152, with *chrIV*:336186::CUT60ter-Pgal-PP7x14-mAIRN-ADH1ter transcribed in **HO** relative to the replisome moving from ARS413	This study
Y167	mAIRN CoD Rnh1 OE	Same as Y152, with *ura3-1*::URA3-Pgal-RNH1-3HA-CYC1ter	This study
Y168	mAIRN HO Rnh1 OE	Same as Y153, with *ura3-1*::URA3-Pgal-RNH1-3HA-CYC1ter	This study
Y169	GAL10orf CoD Rnh1 OE	Same as Y152, with *chrIV*:336186::CUT60ter-Pgal-PP7x14-GAL10orf-CUT60ter transcribed in **CoD** relative to the replisome moving from ARS413; *ura3-1*::URA3-Pgal-RNH1-3HA-CYC1ter	This study
Y156	mAIRN CoD *rnh1rnh201*ΔΔ	Same as Y152, but with *rnh1*Δ::*natMX*; *rnh201*Δ::*hphMX*	This study
Y157	mAIRN HO *rnh1rnh201*ΔΔ	Same as Y153, but with *rnh1*Δ::*natMX*; *rnh201*Δ::*hphMX*	This study
Y163	mAIRN CoD *rnh1rnh201*ΔΔ Rnh1 OE	Same as Y156, but with *natMX*::Pgal-RNH1-3HA-CYC1ter	This study
Y164	mAIRN HO *rnh1rnh201*ΔΔ Rnh1 OE	Same as Y157, but with *natMX*::Pgal-RNH1-3HA-CYC1ter	This study
Y162	mAIRN CoD *sen1-3*	Same as Y153, with SEN1::*sen1-3 (W773A, E774A, W777A)*	This study
Y161	mAIRN HO *sen1-3*	Same as Y152, with SEN1::*sen1-3 (W773A, E774A, W777A)*	This study
Y170	mAIRN HO *sen1-3* Rnh1 OE	Same as Y161, with *ura3-1*::URA3-Pgal-RNH1-3HA-CYC1ter	This study
Y165	mAIRN CoD *mph1*Δ	Same as Y152, with *mph1*Δ::*kanMX*	This study
Y166	mAIRN HO *mph1*Δ	Same as Y153, with *mph1*Δ::*kanMX*	This study
Y173	mAIRN HO *mph1*Δ Rnh1 OE	Same as Y166, with *ura3-1*::URA3-Pgal-RNH1-3HA-CYC1ter	This study
Y174	MAIRN CoD *rpb1-1*	Same as Y152, with RPB1::*rpb1-1 (G4622A)*	This study
Y175	mAIRN HO *rpb1-1*	Same as Y153, with RPB1::*rpb1-1 (G4622A)*	This study
Y176	mAIRN CoD *rpb1-1* *R*nh1 OE	Same as Y174, with *ura3-1*::URA3-Pgal-RNH1-3HA-CYC1ter	This study
Y177	mAIRN HO *rpb1-1* Rnh1 OE	Same as 175, with *ura3-1*::URA3-Pgal-RNH1-3HA-CYC1ter	This study
yCW3	WT	*MATa; his3Δ1; leu2Δ0; ura3Δ0; met15Δ0; LYS2*	This study
yCW4	WT	*MATa; his3Δ1; leu2Δ0; ura3Δ0; met15Δ0; LYS2*	This study
yCW5	WT	*MATa; his3Δ1; leu2Δ0; ura3Δ0; met15Δ0; LYS2*	This study

yCW919	*rnh1Δ*	*MATa; his3Δ1; leu2Δ0; ura3Δ0; rnh1::hygMX*	This study
yCW920	*rnh1Δ*	*MATa; his3Δ1; leu2Δ0; ura3Δ0; rnh1::hygMX*	This study
yCW921	*rnh1Δ*	*MATa; his3Δ1; leu2Δ0; ura3Δ0; rnh1::hygMX*	This study
yCW922	*sen1-1*	*MATa; his3Δ1; leu2Δ0; ura3Δ0; sen1-1:kanMX*	This study
yCW923	*sen1-1*	*MATa; his3Δ1; leu2Δ0; ura3Δ0; sen1-1:kanMX*	This study
yCW924	*sen1-1*	*MATa; his3Δ1; leu2Δ0; ura3Δ0; sen1-1:kanMX*	This study
yCW925	*sen1-1 rnh1Δ*	*MATa; his3Δ1; leu2Δ0; ura3Δ0; rnh1::hygMX; sen1-1:kanMX*	This study
yCW926	*sen1-1 rnh1Δ*	*MATa; his3Δ1; leu2Δ0; ura3Δ0; rnh1::hygMX; sen1-1:kanMX*	This study
yCW927	*sen1-1 rnh1Δ*	*MATa; his3Δ1; leu2Δ0; ura3Δ0; rnh1::hygMX; sen1-1:kanMX*	This study
yCW1036	*G1-RNH1*	*MATa; his3Δ1; leu2Δ0; ura3Δ0; natMX:pSIC1-SIC1(1-315)-RNH1-6xHA:kanMX*	This study
yCW1037	*G1-RNH1*	*MATa; his3Δ1; leu2Δ0; ura3Δ0; natMX:pSIC1-SIC1(1-315)-RNH1-6xHA:kanMX*	This study
yCW1040	*G1-RNH1*	*MATa; his3Δ1; leu2Δ0; ura3Δ0; natMX:pSIC1-SIC1(1-315)-RNH1-6xHA:kanMX*	This study
yCW1035	*sen1-1 G1-RNH1*	*MATalpha; his3Δ1; leu2Δ0; ura3Δ0; sen1-1:hygMX; natMX:pSIC1-SIC1(1-315)-RNH1-6xHA:kanMX*	This study
This study yCW1038	*sen1-1 G1-RNH1*	*MATa; his3Δ1; leu2Δ0; ura3Δ0; sen1-1:hygMX; natMX:pSIC1-SIC1(1-315)-RNH1-6xHA:kanMX*	This study
yCW1039	*sen1-1 G1-RNH1*	*MATa; his3Δ1; leu2Δ0; ura3Δ0; sen1-1:hygMX; natMX:pSIC1-SIC1(1-315)-RNH1-6xHA:kanMX*	This study
yCW1083	*S-RNH1*	*MATalpha; his3Δ1; leu2Δ0; ura3Δ0; natMX:pCLB6-CLB6(1-585)-RNH1-6xHA:kanMX*	This study
yCW1084	*S-RNH1*	*MATa; his3Δ1; leu2Δ0; ura3Δ0; natMX:pCLB6-CLB6(1-585)-RNH1-6xHA:kanMX*	This study
yCW1085	*S-RNH1*	*MATa; his3Δ1; leu2Δ0; ura3Δ0; natMX:pCLB6-CLB6(1-585)-RNH1-6xHA:kanMX*	This study
yCW1080	*sen1-1 S-RNH1*	*MATa; his3Δ1; leu2Δ0; ura3Δ0; natMX:pCLB6-CLB6(1-585)-RNH1-6xHA:kanMX; sen1-1:hygMX*	This study
yCW1081	*sen1-1 S-RNH1*	*MATalpha; his3Δ1; leu2Δ0; ura3Δ0; natMX:pCLB6-CLB6(1-585)-RNH1-6xHA:kanMX; sen1-1:hygMX*	This study
yCW1082	*sen1-1 S-RNH1*	*MATa; his3Δ1; leu2Δ0; ura3Δ0; natMX:pCLB6-CLB6(1-585)-RNH1-6xHA:kanMX; sen1-1:hygMX*	This study
yCW1031	*G2-RNH1*	*MATa; his3Δ1; leu2Δ0; ura3Δ0; natMX:pCLB2-CLB2(1-540)-RNH1-TAP:hisMX*	This study
yCW1032	*G2-RNH1*	*MATa; his3Δ1; leu2Δ0; ura3Δ0; natMX:pCLB2-CLB2(1-540)-RNH1-TAP:hisMX*	This study
yCW1034	*G2-RNH1*	*MATalpha; his3Δ1; leu2Δ0; ura3Δ0; natMX:pCLB2-CLB2(1-540)-RNH1-TAP:hisMX*	This study
yCW1029	*sen1-1 G2-RNH1*	*MATa; his3Δ1; leu2Δ0; ura3Δ0; sen1-1:hygMX; natMX:pCLB2-CLB2(1-540)-RNH1-TAP:hisMX*	This study
yCW1030	*sen1-1 G2-RNH1*	*MATa; his3Δ1; leu2Δ0; ura3Δ0; sen1-1:hygMX; natMX:pCLB2-CLB2(1-540)-RNH1-TAP:hisMX*	This study
yCW1033	*sen1-1 G2-RNH1*	*MATa; his3Δ1; leu2Δ0; ura3Δ0; sen1-1:hygMX; natMX:pCLB2-CLB2(1-540)-RNH1-TAP:hisMX*	This study
B0001 (Cat# 18265017)	*Escherichia coli* DH5alpha	-	Thermo Fischer Scientific

Table S2: Plasmids used in this study

Identifier	Plasmid	Source
K349	pAG25-C60ter-Pgal-14xPP7-mAIRN-ADH1ter	This study
K346	pTL-Pgal-RNH1-3HA-CYC1ter	This study
K350	pCAS9-RPB1-gRNA2	Tsirkas et al. [1]
K351	pCAS9-SEN1-gRNA1	This study
K142	MNase-3xHA	This study

Table S3: Oligonucleotides used in the study.

Name	Sequence (5'-3')
mAIRN5' Frw	tagaggattccgcaaaggaa
mAIRN5' Rev	ttcaccctagcgctgaatct
mAIRN3' Frw	cgagagaggctaagggtgaa
mAIRN3' Rev	acatggtcctgctggagttc
PP7-mAIRN Frw	cgatgtacatggagaattcgag
PP7-mAIRN Rev	cggtgctgtgcttcttg
mAIRN-middle Frw	ttagagcacgagggtgccac
mAIRN-middle Rev	cggatcaggtcgtgtagaaatcctcc
mAIRN-end Frw	aggattcgaagggttctgtg
mAIRN-end Rev	cgtgagcttgcctctctt
PP7-GAL10 Frw	acgaagttattaggtgatatcagatcc
PP7-GAL10 Rev	accaatgtatccagcaccac
PEX19 Frw	ccaagttcggatcattaccatca
PEX19 Rev	tggacgaattagaacagcttgg
ACT1orf Frw	agagttgccccagaagaaca
ACT1orf Rev	ggcttggatggaaacgtaga
TDH3orf Frw	caaggaaaccacctacga
TDH3orf Rev	cgaagatggaagagtgagag
mAIRN Frw	gattccggttgggctgtgattc
mAIRN Rev	gcagtttgcaccctcaggatacc

Spike-in Frw (M13 Frw)	gtaaaacgacggccagt
Spike-in Rev (M13 Rev)	caggaaacagctatgac
mAIRN-3-SB-Frw	gctcagagggttccgagctatcc
mAIRN-3-SB-Rev	gttcatctctcctgtaacatggcac

Table S4: Reagents and commercial kits/assays used in this study

Reagent/Material	Source	Identifier
Anti-HA-Peroxidase, high affinity (3F10)	Roche	Cat# 12013819001; RRID:AB_390917
Anti-RNA Polymerase II CTD Antibody (8WG16)	Millipore	Cat# 05-952; RRID:AB_11213782
Anti-RNA polymerase II CTD repeat YSPTSPS (phospho S2)	Abcam	Cat# ab5095; RRID:AB_304749
Polyclonal RNA polymerase II CTD repeat YSPTSPS (phospho S2)	Abcam	Cat# ab193468; RRID:AB_290555
Goat Anti-Rabbit IgG (H+L), Horseradish peroxidase conjugate	Invitrogen	Cat# G21234; RRID:AB_2536530
Mouse anti-RNA:DNA	Cimprich research group	-
S9.6 antibody	Thermo Fisher Scientific	Cat# MSM1-2006-P1; RRID:AB_3095416
Yeast Alpha-Factor Mating Pheromone	Genscript	Cat# RP01002
Concanavalin A	Sigma-Aldrich	Cat# L7647
Silicon Rhodamine-HALO dye	Tsirkas et al. [1]	N/A
Polyethylene Glycol 3350	Sigma-Aldrich	Cat# P4338
Lithium acetate dihydrate	Sigma-Aldrich	Cat# L6883
Herring Sperm DNA	Promega	Cat# D1816
Lithium Chloride	CarlRoth	Cat# 2312123
EGTA	SantaCruz	Cat# Sc-3593A
Tris Base	ChemCruz	Cat# sc-3715B

Zymolyase 20T	Carl ROTH	Cat# 9329.1
XmaI	New England Biolabs	Cat# R0180
PmlI	New England Biolabs	Cat# R0532
EcoRI-HF	New England Biolabs	Cat# R3101
BsrGI-HF	New England Biolabs	Cat# R3575
HindIII-HF	New England Biolabs	Cat# R3104
SacI-HF	New England Biolabs	Cat# R3156
XbaI	New England Biolabs	Cat# R0145
rCutsmart	New England Biolabs	Cat# B6004
Thermostable RNase H	New England Biolabs	Cat# M0523
Guanidine-HCl	Promega	Cat# H5381
β-mercaptoethanol (β-ME)	Sigma-Aldrich	Cat# 07604-100ml
Formaldehyde	Thermo Fisher Scientific	Cat# 28908
Pierce™ Protein A/G Agarose beads	Thermo Fisher Scientific	Cat# 20421
Pierce Protein A Magnetic Beads	Thermo Fisher Scientific	Cat# 10001D
Deoxycholic acid	Santa Cruz	Cat# sc-214865A
Triton X-100	Sigma-Aldrich	Cat# X100-100ML
Spermidine	Sigma-Aldrich	Cat# S0266-1G
Spermine	Sigma-Aldrich	Cat# S3256
SYBR Safe DNA Gel Stain	Invitrogen	Cat# S33102
Ammonium Acetate	SERVA	Cat# 39750.01
Hydrochloric Acid 5N	AppliChem	Cat# 182109.1211
Sodium Hydroxide	Sigma-Aldrich	Cat# S5881
Sodium Chloride	Supelco	Cat# 1.06404.5000

DNase I	New England Biolabs	Cat# M0303S
RNase A	Thermo Fisher Scientific	Cat# EN0531
Proteinase K	SERVA	Cat# 33756
Phenol:chlorophorm:isoamyl alcohol (25:24:1; v/v)	Invitrogen	Cat# 15593-031
OmniPur Phenol:Chloroform:Isoamyl Alcohol, 25:24:1	Millipore	Cat# 6805-100ML
Glycogen	Thermo Fisher Scientific	Cat# AM9510
Igepal CA-630	Sigma-Aldrich	Cat# I3021-100ML
Halt Protease and Phosphatase Inhibitor Cocktail (100x)	Thermo Fisher Scientific	Cat# 78446
BSA Fraction V	Sigma-Aldrich	Cat# 10735078001
Tween-20	Kraft	Cat# 21440.2000
NuPAGE 4-12% Bis-Tris Protein Gels 1.5 mm	Thermo Fisher Scientific	Cat# NP0336BOX
Immobilon Transfer Membrane PVDF	Millipore	Cat# IPVH00010
SuperSignal™ West Pico PLUS Chemiluminescent Substrate	Thermo Fisher Scientific	Cat# 34580
iTaq Universal SYBR Green Supermix	Bio-Rad	Cat# 1725121
SuperScript III First-Strand Synthesis System	Thermo Fisher Scientific	Cat# 18080-051
RadPrime DNA Labeling System	Invitrogen	Cat# 18428-011
NEBNext® Ultra™ II DNA Library Prep	New England Biolabs	Cat# E7103
μ-Slide 8 Well Uncoated	Ibidi	Cat# 80821

Table S5: Software and algorithms used in this study

Software/Algorithm	Source	Identifier
ImageJ	NIH	https://imagej.net/ij/index.html
Zen (version 3.0 blue edition)	Zeiss	https://www.micr-shop.zeiss.com/ en/us/softwarefinder/#select-soft-ware

Matlab (version 2019b)	MathWorks	https://www.mathworks.com/products.html?s_tid=gn_ps
Transcriptomatic5	This study	10.5281/zenodo.15601453
Python (versions 2.7 and 3.8)	Python	https://www.python.org
PyCharm (2020.2)	JetBrains	https://www.jetbrains.com/pycharm/
R v4.1.2	R core Team	https://www.r-project.org
GraphPad Prism 9	GraphPad Software	https://www.graphpad.com

Table S6: Median replication time and cell number of yeast strains for live-cell imaging experiments

Yeast Strain and condition	Median Replication time (min)	Cell number
mAIRN CoD Uninduced	17.9	70
mAIRN CoD Induced	17.5	85
mAIRN HO Uninduced	17.4	69
mAIRN HO Induced	17.6	71
mAIRN CoD Rnh1 OE Induced	18.1	95
mAIRN HO Rnh1 OE Induced	17.7	69
GAL10orf CoD Rnh1 OE Induced	17.0	96
mAIRN CoD *rnh1rnh201ΔΔ* Uninduced	16.8	61
mAIRN CoD *rnh1rnh201ΔΔ* Induced	18.8	75
mAIRN HO *rnh1rnh201ΔΔ* Uninduced	14.3	62
mAIRN HO *rnh1rnh201ΔΔ* Induced	17.0	67
mAIRN CoD *rnh1rnh201ΔΔ* Rnh1 OE Induced	17.3	53
mAIRN HO *rnh1rnh201ΔΔ* Rnh1 OE Induced	15.0	57
mAIRN CoD *sen1-3* Uninduced	16.3	50
mAIRN CoD *sen1-3* Induced	16.8	50
mAIRN HO *sen1-3* Uninduced	17.3	59
mAIRN HO *sen1-3* Induced	19.4	61
mAIRN HO *sen1-3* Rnh1 OE Induced	16.7	55
mAIRN CoD *mph1Δ* Induced	17.1	50

mAIRN HO *mph1*Δ Induced	20.2	66
mAIRN HO *mph1*Δ Rnh1 OE Induced	15.7	32
mAIRN CoD *rpb1-1* Induced	17.1	63
mAIRN HO *rpb1-1* Induced	17.1	70
mAIRN CoD *rpb1-1* Rnh1 OE Induced	19.1	54
mAIRN HO *rpb1-1* Rnh1 OE Induced	20.1	66

REFERENCES

1. Tsirkas I, Dovrat D, Thangaraj M et al. Transcription-replication coordination revealed in single live cells. *Nucleic Acids Res* 2022;**50**:2143-56.
